# Exploration behavior after reversals is predicted by STN-GPe synaptic plasticity in a basal ganglia model

**DOI:** 10.1016/j.isci.2023.106599

**Published:** 2023-04-11

**Authors:** Oliver Maith, Javier Baladron, Wolfgang Einhäuser, Fred H. Hamker

**Affiliations:** 1 Department of Computer Science, Chemnitz University of Technology, Chemnitz, Germany; 2 Departamento de Ingeniería Informática, Universidad de Santiago de Chile, Santiago, Chile; 3 Institute of Physics, Chemnitz University of Technology, Chemnitz, Germany

**Keywords:** Behavioral neuroscience, Systems neuroscience

## Abstract

Humans can quickly adapt their behavior to changes in the environment. Classical reversal learning tasks mainly measure how well participants can disengage from a previously successful behavior but not how alternative responses are explored. Here, we propose a novel 5-choice reversal learning task with alternating position-reward contingencies to study exploration behavior after a reversal. We compare human exploratory saccade behavior with a prediction obtained from a neuro-computational model of the basal ganglia. A new synaptic plasticity rule for learning the connectivity between the subthalamic nucleus (STN) and external globus pallidus (GPe) results in exploration biases to previously rewarded positions. The model simulations and human data both show that during experimental experience exploration becomes limited to only those positions that have been rewarded in the past. Our study demonstrates how quite complex behavior may result from a simple sub-circuit within the basal ganglia pathways.

## Highlights

• A novel 5-choice reversal learning task requiring exploration has been designed
• Exploration in human participants is biased toward past, frequently rewarded responses
• A synaptic plasticity rule in the STN-GPe loop biases exploration after rule changes
• A model of the basal ganglia predicts human exploration behavior

## Graphical abstract

**Figure:**
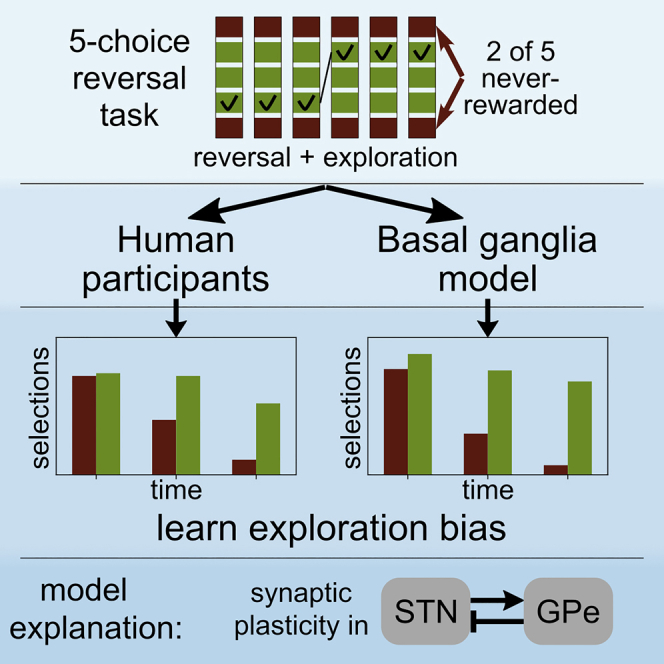

## Introduction

The ability of humans to quickly adapt their behavior to changes in the environment is often being considered a result of rather high-level cognitive processes. It requires to suppress well learned behaviors and to find alternatives. Cognitive and behavioral flexibility engages large brain networks including the orbitofrontal cortex,^1^^,^^2^^,^^3^^,^^4^ the dorsal anterior cingulate cortex,^4^^,^^5^ as well as the basal ganglia,^5^^,^^6^^,^^7^^,^^8^ and it is impaired in various neurological disorders such as Parkinson’s disease, schizophrenia, autism, and amnesia.^9^^,^^10^^,^^11^^,^^12^^,^^13^

A widely used experimental task for studying behavioral flexibility is reversal learning.^2^^,^^14^^,^^15^^,^^16^^,^^17^ Tasks require participants to associate a stimulus, location, or response with reward, but as stimulus-reward contingencies are changed repetitively during the experiment, they aim to test for the participants’ behavioral flexibility. Classical reversal learning tasks^14^^,^^18^^,^^19^ operate with only two choices. In this case, the withdrawal of the previously rewarded choice and the selection of the remaining choice (i.e., the “reversal”) is sufficient for optimal behavior, and no exploration is required. Even though some tasks allow for multiple choices, previous studies did not focus on exploration behavior.^20^^,^^21^^,^^22^ Our aim is to investigate if and how humans may use previously learned associations to guide exploration. Thus, we designed a 5-choice reversal learning task with alternating position-reward contingencies to test if exploration behavior depends on previous stimulus-reward associations. “Reversal” here refers to a broader view in that the reward is not necessarily switched back to the most recent rewarded position but to one of a small subset of positions. “Exploration” refers to the need of finding the newly rewarded response among several possible responses with uncertain outcome, which deviates slightly from a more typical usage of the term in the context of the exploration-exploitation dilemma for finding a balance between collecting new information but still performing a required task.^23^ In reinforcement-learning nomenclature, a biased exploration in our task corresponds to different values for the possible responses (only during the exploration phase).

Although the basal ganglia have been recognized to play a role in reversal learning,^6^^,^^7^^,^^8^^,^^9^^,^^12^^,^^18^^,^^19^^,^^24^^,^^25^^,^^26^^,^^27^^,^^28^^,^^29^ several questions are yet to be answered. The understanding of the function of the basal ganglia has been guided by the distinction between the direct and indirect basal ganglia pathways.^30^^,^^31^^,^^32^^,^^33^ The former promotes responses by reducing the baseline inhibition exerted on the projection targets of the basal ganglia. The latter produces the contrary effect by increasing the inhibition. A third pathway, the hyperdirect pathway,^34^ is associated with general stopping or pausing of responses.^35^^,^^36^^,^^37^^,^^38^^,^^39^^,^^40^^,^^41^ In reversal learning tasks the indirect or hyperdirect pathway may suppress the previously rewarded response, and the direct pathway may establish and maintain the newly rewarded response. Once participants understand the concept of this classical reversal learning task, no learning, but rather a switch to the alternative choice is required. Our 5-choice reversal learning task with alternating position-reward contingencies, however, requires not only suppressing the previously rewarded choice, but also an active exploration to find the newly rewarded one.

Although the three major pathways in the basal ganglia are well established, they are less independent than originally thought. First, the hyperdirect pathway can also indirectly decrease the inhibition in the output nuclei of the basal ganglia through the connections between the subthalamic nucleus (STN) and the external globus pallidus (GPe).^42^ Accordingly, Isoda and Hikosaka^43^ found STN neurons associated with both, stopping and facilitating responses during behavioral switching. The GPe, in turn, also projects back to the STN, creating a bidirectional circuit whose function is still unexplained. Furthermore, Wang et al.^19^ have recently shown that optogenetic stimulation of striatal neurons of the indirect pathways during reversal learning appears to increase thalamic activity. This contradicts the classical view that the indirect pathway only suppresses the previously learned response by inhibiting the thalamus.

Accordingly, using a computational model of the basal ganglia, we recently hypothesized that the indirect pathway, activated by unrewarded responses, not only suppresses previously rewarded responses but also additionally activates the bidirectional STN-GPe circuit^44^ in order to facilitate the exploration of some alternative responses against others. This newly proposed function of the recurrent STN-GPe circuit is particularly interesting in a reversal learning scenario where multiple alternative responses need to be explored. In this previous modeling study,^44^ we demonstrated that different STN → GPe connectivity patterns lead to different exploration behavior after a reversal.

In the present study, we derive a learning rule that allows the STN → GPe connections to accumulate prior experience by means of synaptic plasticity. While synaptic plasticity in the basal ganglia is often studied in the striatum, many findings do also indicate synaptic plasticity in the bidirectional STN-GPe circuit,^45^^,^^46^^,^^47^^,^^48^^,^^49^^,^^50^^,^^51^ including dopaminergic innervation that affects synaptic plasticity.^52^^,^^53^ This extended model now allows us to investigate and predict the putative influence of STN → GPe synaptic plasticity on exploration behavior in a 5-choice reversal learning task with alternating position-reward contingencies, without a prior definition of a particular STN → GPe connectivity. Our simulations served as predictions which were tested in experiments with human participants. We decided to study exploration behavior in a saccade task as the basal ganglia are directly involved in the control of eye movements by substantia nigra pars reticulata (SNr) to superior colliculus (SC) projections^43^^,^^54^^,^^55^^,^^56^ and because rapid saccadic responses minimize the influence of planning and strategic behavior dominated by other brain areas such as the prefrontal cortex.

## Results

### 5-Choice reversal learning task with alternating position-reward contingencies

**Figure 1:**
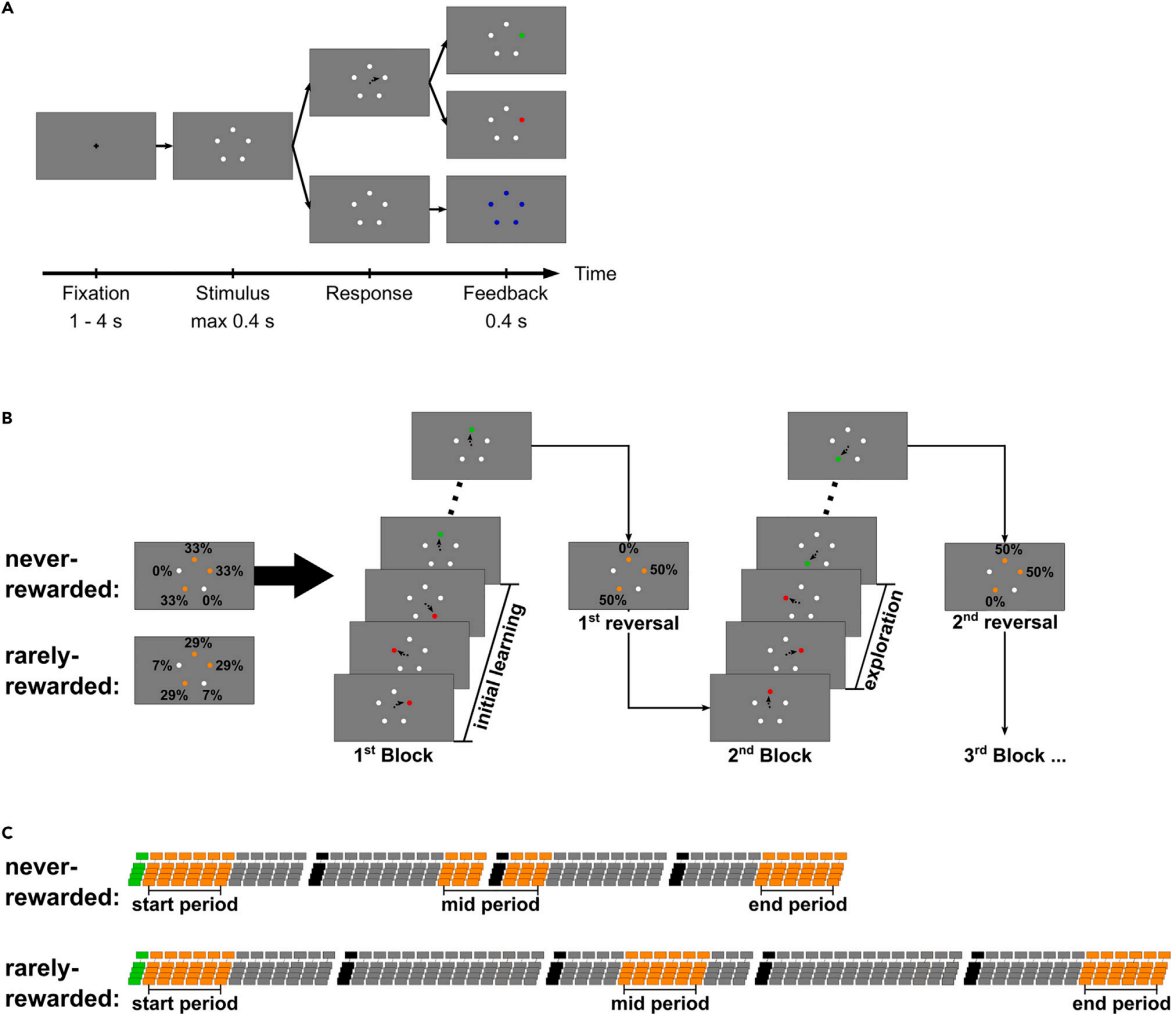
Structure of the 5-choice reversal learning task (A) Single trial of the task. After a central fixation, five identical, small white circles at unique positions arranged on an imaginary circle are presented. Participants are required to saccade to one of the five positions within 400 ms. If a saccade response occurs, a positive-reward feedback (selected circle → green) or a negative-reward feedback (selected circle → red) is given. Without a response, a negative-reward feedback is given after 400 ms (all circles → blue). (B) Initial learning phase and first reversal learning phase of the never-rewarded experiment. The first block contains the initial learning phase; all following blocks start with a reversal thus contain an exploration. Unbeknownst to the participants, three positions are selected at the beginning of each experiment (highlighted here in orange) that are frequently rewarded during the different blocks of the task. The remaining two positions are never rewarded in the never-rewarded experiment and rarely rewarded in the rarely-rewarded experiment. Throughout a block, the same position is rewarded in all trials. After some consecutive rewarded trials or after a maximum number of trials (see method details), a new, different, rewarded position for the next block is randomly determined (= reversal, indicated only in this figure not during task). The participants become aware of the reversal, when they receive negative-reward feedback for their previously rewarded choice. With the selection of an alternative position the exploration phase starts. After some exploration trials the newly rewarded position is discovered, ending the exploration phase. (C) Different periods of the experiment. 48 blocks are conducted (60 for the model) in the never-rewarded experiment and 70 blocks in the rarely-rewarded experiment. The first block contains the initial learning phase (highlighted in green). In our analysis, we assign blocks to three different periods (highlighted in orange); the start period, mid period, and end period. The start period contains the 6 initial exploration phases (from blocks 2 to 7), the mid period contains the 6 intermediate exploration phases, and the end period contains the 6 final exploration phases (only one exploration phase per period was used for the model). The data of the blocks following a break (every 12 blocks in never-rewarded experiment, every 14 blocks in rarely-rewarded experiment, highlighted black) are excluded from the analysis.

We designed a novel 5-choice reversal learning task (Figure 1), to which we subjected human participants and our basal ganglia model alike. In each trial, the agent (human or model) was asked to select one out of five positions (Figure 1A), only one of which was rewarded. For several consecutive trials (hereafter referred to as “block”), the same position was rewarded, then—without informing the agent—a different block started, the rule changed, and a different position was rewarded. We conducted two different versions of this task ($N_{\text{model}}=60$, $N_{\text{participants}}=10$ for both). In the first version (referred to as never-rewarded experiment), the rewarded position was determined from only three out of the five response options (without direct repetitions). Thus, two positions were never rewarded (except for an initial task familiarization phase, where reward was uniformly allocated). In the second version (referred to as rarely-rewarded experiment), these two positions were rarely rewarded (four times less often than the frequently rewarded positions). Human participants and model were not informed about these rules but merely instructed to maximize their reward. By design, “reversal” refers to a rule change that in our task implies an exploration phase (see Figure 1B). Models and human participants had to continually adapt to the alternating position-reward contingencies.

For the human participants, the task was implemented as choice between five saccade targets, each item uniquely identified by its position on an imaginary circle around central fixation. On disappearance of the fixation cross, participants had to shift gaze as quickly as possible to one of the positions (Figure 1A), as they only could gain reward, if their gaze reached the rewarded position with the first saccade within 400 ms after the offset of the fixation cross.

**Figure 2:**
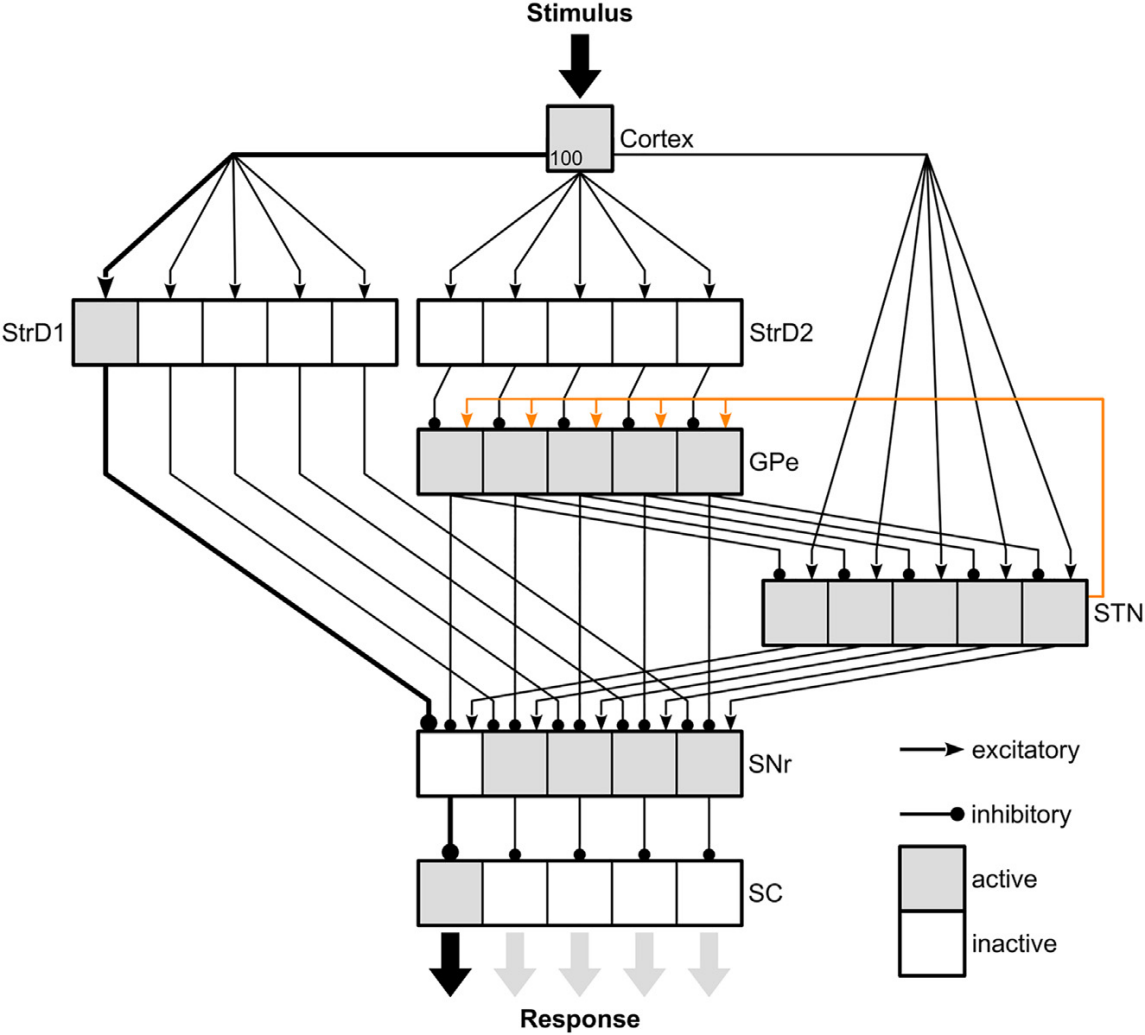
Model overview The model is composed of neural patches (squares), each with a population of 100 Izhikevich^68^ spiking neurons and connected to each other according to the major basal ganglia pathways. The presentation of the stimulus requiring the selection of one of five positions is simulated by activating the 100 cortical neurons which randomly emit spikes following a Poisson distribution. We pre-defined 5 individual sub-channels, each encoding the selection of one position. For the STN → GPe projection, only the efferent projection of the rightmost STN sub-population is shown for clarity. The cortical projections to the direct pathway (StrD1 → SNr), to the indirect pathway (StrD2 → GPe → SNr), and to the hyperdirect pathway (STN → SNr) underlie dopamine-modulated synaptic plasticity. Plasticity in the STN → GPe projection (highlighted in orange) is also modulated by dopamine. Plasticity in the cortical projections causes sub-channel specific weight changes which allow the model to learn to excite or suppress the selection of a particular position following a stimulus presentation. The plasticity in the STN → GPe projection regulates the inhibitory effect of the STN on the SNr via the GPe, which is particularly relevant for exploration after reversals. For simplicity, local projections in StrD1 and the SC, the cortex → SC projection, the feedback from SC to StrD1 and StrD2 are not shown. The selected position of the model is readout from the SC by accumulating the activity of its sub-populations. By reducing the constant inhibition from SNr onto SC, the SC can get active and a model response can occur. Following a stimulus presentation, the activity of one sub-population of the SNr is reduced via the direct pathway and increased (disinhibited) via the indirect pathway. The hyperdirect pathway provides surround inhibition (globally excite SNr) but also inhibits SNr neurons of alternative responses indirectly (STN → GPe → SNr). The changes in activity during the leftmost response are indicated in a simplified manner. StrD1 – D1 dopamine receptor expressing striatal projection neurons, StrD2 – D2 dopamine receptor expressing striatal projection neurons, GPe – external globus pallidus, STN – subthalamic nucleus, SNr – subtantia nigra pars reticulata, SC – superior colliculus.

Our model includes the main neuronal compartments and pathways of a basal ganglia circuit (Figure 2), each represented by separate populations of single-compartment spiking neurons and provides a selected position as output following stimulus presentation. All cortical projections to the striatum and the STN underlie synaptic plasticity, allowing the model to learn stimulus-response associations using a reward signal encoded in the dopamine level. The crucial innovation with respect to previous models is the dopamine-modulated plasticity in the STN → GPe projection, which, as we will show below, is particularly relevant for exploration (see model explanation).

Before conducting experiments with human participants, we obtained a model prediction with our basal ganglia model on the never-rewarded experiment (see Figure S1). We found that in the subsequent experiment, the participants’ behavior confirmed the prediction. This successful prediction, however, left open whether humans (and the model) could also deal with situations in which two positions are rarely (rather than never) rewarded. We therefore conducted the rarely-rewarded experiment with model and a new group of participants and observed slight discrepancies between the model prediction and experimental data. To account also for the new observations, we slightly revised the model and report the results of the revised model in the main text. For full transparency, the differences between the original and the current model simulations are described in method details and Table 1. Thus, our study demonstrates a typical multi-step validation process, including initial model prediction, subsequent experimental validation, extension of the experiment to a related setting, and finally model adjustment to capture the original and the refined setting, thereby incrementally increasing the model’s scope.

**Table 1:** Parameters for the plastic STN→GPe projection

Parameter	Value
$\tau_{A}$ [ms]	50
$\tau_{\bar{A}}$ [ms]	10^4^
$\tau_{w}$ [ms]	$\frac{50}{6}\times 10^{6}$
ΔA	0.001
ϵ	10
$w_{\max}$ [nS]	0.00145
$\tau_{DA}$ [ms]	19
$w_{INIT}$ [nS]	0.00063

The units of the variables are given in brackets. For the original model simulations, we used a larger $\tau_{w}=\frac{50}{3}\times 10^{6}$ and a smaller ϵ=8. Compared to the original model, we therefore increased the learning speed to compensate for the smaller average reward signal (which decreases the learning speed). However, no exhaustive parameter adjustment was made, which could certainly be used to achieve an even better fit to the human data but was not necessary to clearly demonstrate our main points.

### Overall human performance

#### Never-rewarded experiment

A familiarization phase was followed by 48 blocks in the never-rewarded experiment (see Figure 1C). A block ended on average after 8 consecutive rewarded trials or a maximum of 14 trials. Besides a break after every 12 blocks, there was no explicit indication of transitions between blocks. Thus, participants could not anticipate the reversals.

**Figure 3:**
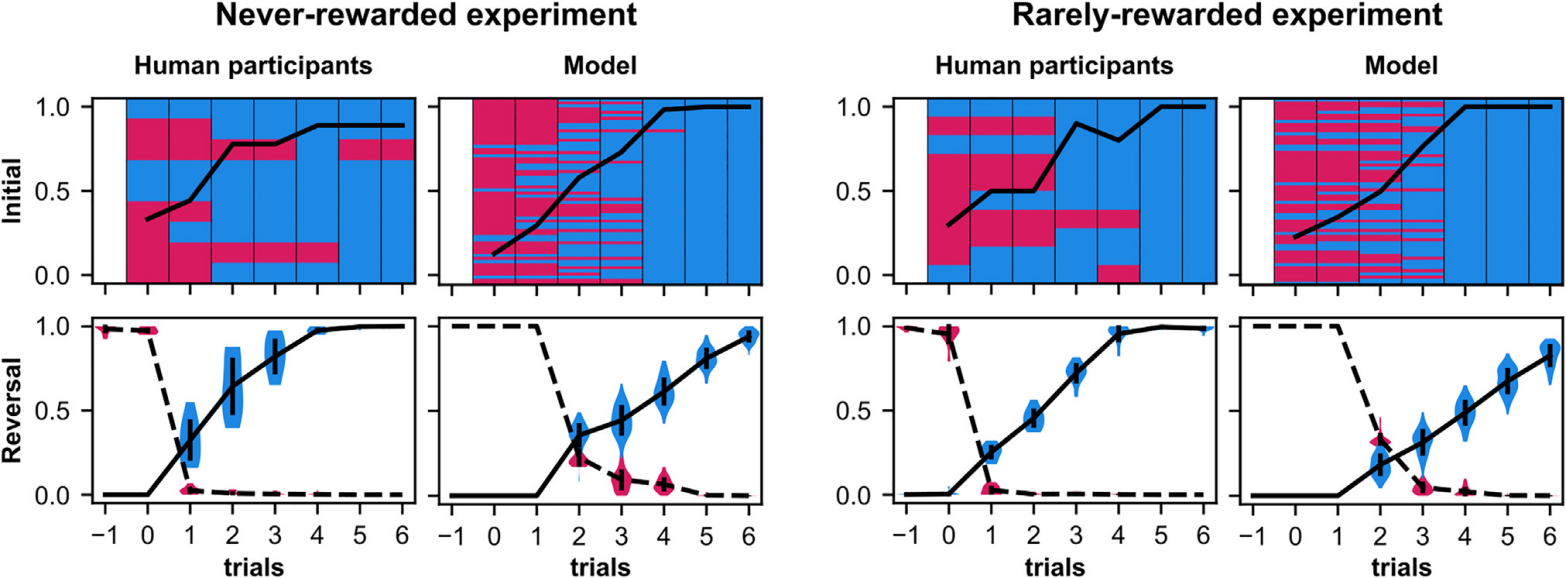
Human and model performance during the first trials of the initial learning phase (beginning of the first block) and reversal learning phases (beginning of all later blocks following reversals) for the never- and rarely-rewarded experiment For the initial learning phase, the black line is the average probability of choosing the rewarded position. The selections of each individual participant/simulation are displayed in the background (red = unrewarded, blue = rewarded). Each row corresponds to a different participant/simulation. For the reversal learning phases, the average probability of choosing the rewarded position (solid line) and the average probability of choosing the previously rewarded position (dashed line) are shown. The distributions over the whole data range of the participants/simulations are indicated as violin plots and the standard deviation is given by the vertical black lines (not visible for some coordinates because the variance is too small). During reversal learning phases the probability of choosing the previously rewarded position quickly drops. The model needs more trials to disengage from the previously rewarded choice than the human participants. Plotted lines are averaged over 10 participants (9 for initial learning of never-rewarded experiment due to unsuccessful completion of one participant, thus only 9 rows are shown) and 60 simulations for both versions of the experiment. The data are aligned on the first trial of each block (trial 0). For the reversal learning phases, also the trial before the reversal is shown. Data for the original model is shown in Figure S2.

On average, in 91.11% (SD = 7.95%) of blocks, participants managed to select the rewarded position at least 7 times in a row. Thus, participants quickly adapted their behavior to select the newly rewarded position (Figure 3). On average, participants made 2.00 (SD = 2.05) unrewarded selections before their selection converged to the rewarded position in the initial learning phase and 2.24 (SD = 0.39) unrewarded selections during the reversal learning phases (including the first trial after reversal).

The average response time (duration between stimulus onset and when the gaze reaches the target area) did not differ between consecutive rewarded trials and exploration trials (two-tailed t-test for dependent samples: *t*(9) = −0.55, p = 0.595, $d_{z}$ = −0.18, 95% CI -3.91 to 2.31; response time all trials: *M* = 174 ms, SD = 14 ms). However, the presence of timeouts (response slower than 400 ms) exclusively during exploration trials (*M* = 1.73%, SD = 1.27% of exploration trials) indicates some uncertainty induced by negative-reward feedback.

#### Rarely-rewarded experiment

In the rarely-rewarded experiment, participants went through 70 blocks after a familiarization phase (see Figure 1C). Breaks occurred after every 14 blocks.

On average, in 86.97% (SD = 5.43%) of blocks, participants managed to select the rewarded position at least 7 times in a row (Figure 3). On average, participants made 2.00 (SD = 1.55) unrewarded selections before their selection converged to the rewarded position in the initial learning phase and 2.63 (SD = 0.16) unrewarded selections during the reversal learning phases.

The average response time did not differ between consecutive rewarded trials and exploration trials (two-tailed t-test for dependent samples: *t*(9) = 0.49, p = 0.638, $d_{z}$ = 0.16, 95% CI -1.37 to 2.17; response time all trials: *M* = 172 ms, SD = 12 ms). Also in the rarely-rewarded experiment, the timeouts exclusively occur during exploration trials (*M* = 2.97%, SD = 1.70% of exploration trials).

### Overall model performance

In all simulations conducted (both versions of the experiment), the model was able to adapt its response to the alternating position-reward contingencies and selected the rewarded position 15 times consecutively during all blocks (within a maximum of 30 trials).

The model’s ability to adapt to alternating reward contingencies relies on the plasticity of cortico-striatal and cortico-subthalamic projections (see Figure S3). The strength of synapses to D1-type cells in the striatum (direct pathway) increases in a Hebbian manner in case of a positive reward prediction error while those synapses to D2-type cells (indirect pathway) decrease and vice versa in case of a negative-reward prediction error, similar to observations by Shen et al.^57^ The cortico-subthalamic synapses follow the D1-type learning rule. Thus, the direct pathway promotes rewarded responses, the indirect pathway suppresses unrewarded responses, and the hyperdirect pathway provides surround suppression of alternative responses during rewarded trials, consistent with previous modeling work.^44^^,^^58^

#### Never-rewarded experiment

In the never-rewarded experiment, the model completed 60 blocks. It made an average of 2.27 (SD = 1.39) unrewarded selections until its selection converged to the rewarded position in the initial learning phase. During reversal learning phases, it made significantly more unrewarded selections than the human participants (*M* = 3.84, SD = 0.24, two-tailed Welch’s t-test, *t*(68) = 11.92, *p* < 0.001, *d* = 5.98, 95% CI 1.41 to 1.78) (Figure 3).

#### Rarely-rewarded experiment

In the rarely-rewarded experiment, the model completed 70 blocks. It also made a similar number of unrewarded selections as the human participants in the initial learning phase (*M* = 2.15, SD = 1.49) but significantly more unrewarded selections than the human participants in the reversal learning phases (*M* = 4.51, SD = 0.30, two-tailed t-test for independent samples, *t*(68) = 18.99, *p* < 0.001, *d* = 6.55, 95% CI 1.69 to 2.08) (Figure 3). In particular, the model is slower in disengaging from the previously rewarded response than the participants. Since the model does not explicitly disengage from unrewarded responses as participants do (it learns continuously by adapting its plastic weights as mentioned before), and sometimes repeats the same unrewarded response, we combine multiple selections of the same unrewarded response into one selection in the following analyses (for both the model and participants).

### Human and model exploration behavior

**Figure 4:**
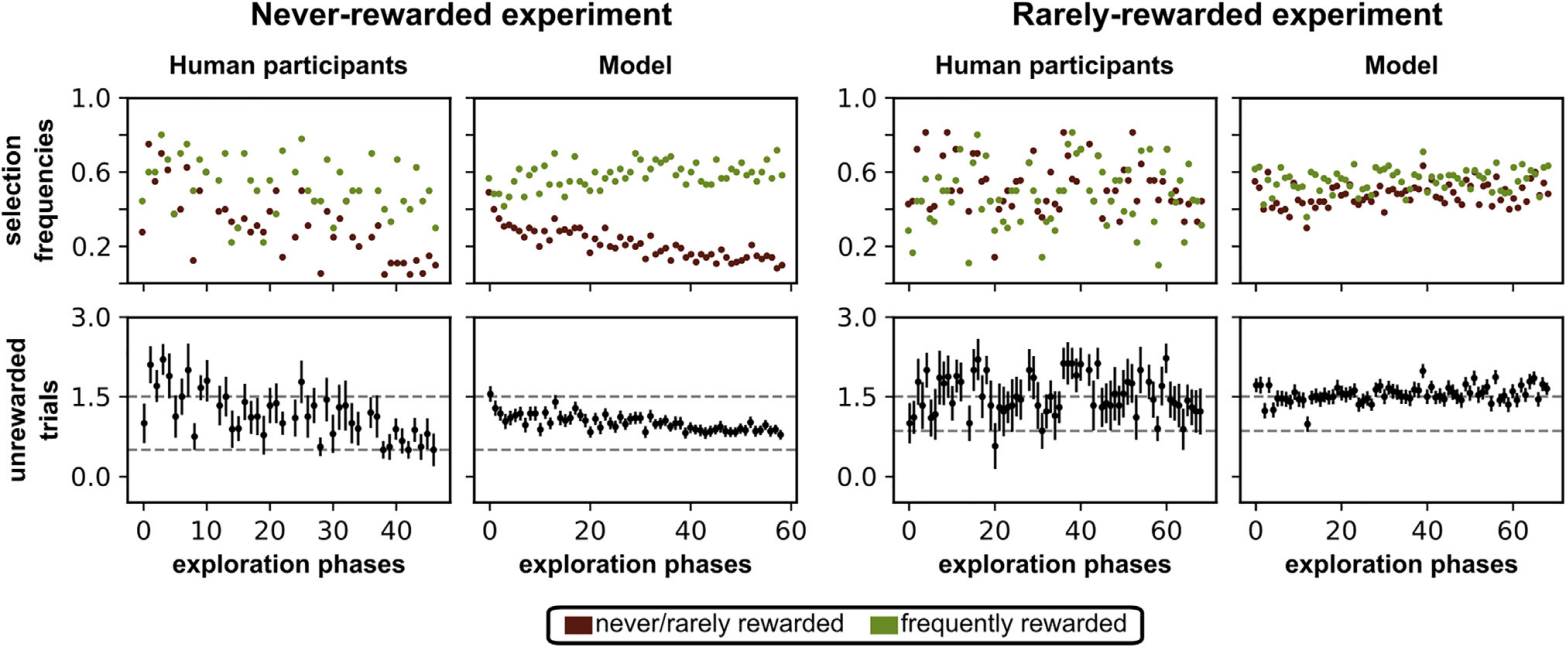
Selection frequencies of unrewarded exploration trials of human participants and the model for the never- and rarely-rewarded experiment After a reversal is detected, participants and models explore alternative responses and make two type of unrewarded responses: they either select a position that is never/rarely rewarded during the task (brown dots) or select a position that is not currently rewarded but frequently rewarded during the task (green dots). In the first row, the dots show the frequency of these two unrewarded response types for each exploration phase of the task averaged over participants/simulations (see quantification and statistical analysis for quantification details). In the never-rewarded experiment, for both the model and the human participants, the frequency of never-rewarded positions decreases with increasing experience during the experiment. In the rarely-rewarded experiment, there is no decrease in the frequency of the rarely rewarded positions. The bottom row shows the average number of unrewarded exploration trials for each exploration phase, with the standard error of the mean as error bars. The dashed lines show the expected average numbers of unrewarded exploration trials for an ideal observer (see method details) showing an exploration bias toward frequently rewarded positions (never-rewarded experiment: 0.5, rarely-rewarded experiment: 0.86) or not (1.5). In the never-rewarded experiment, both the model and the human participants reduce the average number of unrewarded exploration trials to almost 0.5. In the rarely-rewarded experiment, there is no such reduction. The data were obtained from 60 simulations and 10 participants for both versions of the experiment.

As our main focus is on comparing the human and model exploration behavior, we analyzed which responses occurred during unrewarded exploration trials (Figure 4). To obtain comparable selection frequency distributions, we divided the data from the 60 simulations and the data from the 10 participants of each version of the experiment into three periods that reflect the progress of learning: start (60 initial exploration phases), mid (60 intermediate exploration phases), and end (60 final exploration phases) (see Figure 1C). If the participants or the model do not consider previous position-reward contingencies during exploration, they should explore all positions randomly, except of the previously rewarded one. If they, however, learned to particularly explore the three frequently rewarded positions, they would find the new rule on average in less trials. Hence, we analyzed the number of unrewarded exploration trials as a measure of (implicit) learning.

#### Never-rewarded experiment

##### Exploration bias is learned

For human participants, neither in the start period (${\tilde{\chi }}^{2}$ (1) = 0.29, p = 0.589, $\alpha_{corrected}$ = $.01\bar{6}$, Bonferroni corrected for three tests) nor in the mid period (${\tilde{\chi }}^{2}$ (1) = 3.47, p = 0.062) could we find any evidence for differences in selection frequency between the never-rewarded and frequently rewarded positions. In the end period, the selection frequencies differ significantly (${\tilde{\chi }}^{2}$ (1) = 13.34, *p* < 0.001), indicating that participants avoid selecting the two never-rewarded positions (Figure 4). Although participants occasionally search clockwise or counterclockwise due to the circular arrangement of the items (see Figure S4), these explicit exploration rules do not prevent the exploration bias from being learned.

The development of the exploration bias was also predicted by our model. The model does not show significant differences in selection frequency between the never-rewarded and frequently rewarded positions during the start period (${\tilde{\chi }}^{2}$ (1) = 0.32, p = 0.572, $\alpha_{corrected}$ = $.01\bar{6}$, Bonferroni corrected for three tests). However, for the mid (${\tilde{\chi }}^{2}$ (1) = 16.67, *p* < 0.001) and end period (${\tilde{\chi }}^{2}$ (1) = 20.51, *p* < 0.001) the frequencies differ significantly. As outlined below (model explanation), the avoidance of the never-rewarded positions is a result of the learned exploration bias toward previously rewarded positions by the STN-GPe circuit.

##### Unrewarded exploration trials decrease

The number of unrewarded exploration trials made by the human participants was also well predicted by our model (Figure 4, bottom). For both the model and the participants, the average number of unrewarded exploration trials decreases. It appears to decrease from about 1.5 to about 0.5. These values are to be expected for an ideal observer who considers all (resulting on average in 1.5 unrewarded exploration trials) or only the frequently rewarded responses (resulting on average in 0.5 unrewarded exploration trials) during exploration phases (see method details for more details). To quantify the learning rate, we fitted an exponential function to the number of unrewarded exploration trials of each participant/model simulation and obtained a half-life for each fit. Half-lives were further transformed to obtain a learning rate which ranges from −1 to 1 (−1 – fast increase, 0 – no change, 1 – fast decrease, see quantification and statistical analysis for details).

For both the model (*M* = 0.50, SD = 0.30) and the participants (*M* = 0.46, SD = 0.39), the average obtained learning rate is significantly larger than zero (one-tailed t-test for one sample; model: *t*(59) = 12.52, *p* < 0.001, *d* = 1.63, 95% CI 0.42 to 0.58; participants: *t*(9) = 3.48, p = 0.003, *d* = 1.16, 95% CI 0.17 to 0.74). Thus, the number of unrewarded exploration trials significantly decreases. Further, we did not find a significant difference between the learning rate of the model and the human participants (two-tailed t-test for independent samples: *t*(68) = 0.37, p = 0.714, $d_{z}$ = 0.13, 95% CI -0.18 to 0.26).

##### Exploration bias develops continuously

**Figure 5:**
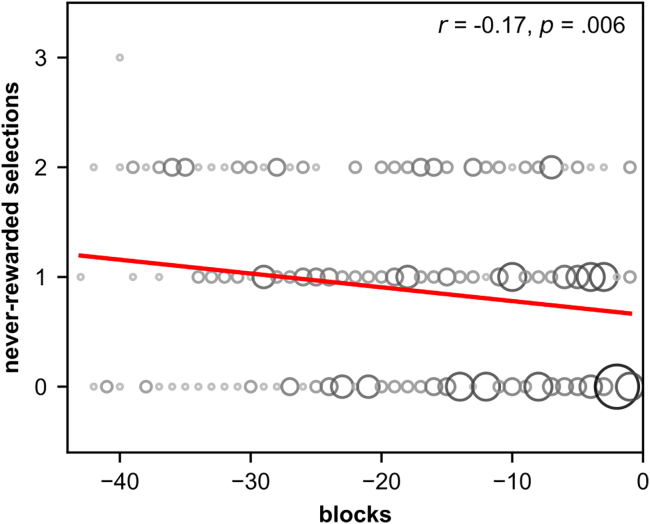
Number of selections of never-rewarded positions by participants prior to the block in which a never-rewarded position was selected for the last time (block 0) For each block the number of selections of never-rewarded positions made by the participants is shown. The number of data points at specific coordinates is represented by the size and saturation of the corresponding circle. The data are taken from 10 participants, thus maximally 10 data points per block are shown. Earlier blocks include fewer points because the block number in which a never-rewarded position was selected for the last time differs between participants, and only data from participants with enough prior blocks can be shown for early blocks. The red line indicates the result of a linear regression. The Spearman correlation between blocks and the number of selections of never-rewarded positions is shown on the top right corner. The data indicates that the number of selections of never-rewarded positions decreases gradually.

We further investigated whether human participants developed the exploration bias suddenly or continuously during the task. A sudden switch from “no bias” to “active bias” would speak for explicit learning and the usage of a higher cognitive rule, whereas a continuous progression toward the bias would rather speak for implicit learning as in the basal ganglia model. Different participants developed the exploration bias at different times in the experiment. However, when we align the data to the block in which a participant has chosen for the last time a position that is never rewarded (see Figure S5), we obtain an across participant estimate of the progress toward the exploration bias (Figure 5). Since the selection frequency of never-rewarded positions decreases throughout the prior blocks (Spearman correlation, *r*(258) = −0.17, p= 0.006, 95% CI -0.29 to −0.05), our data indicate a continuous progress toward the bias. Thus, we can rule out that participants make a sudden, explicit switch from “no bias” to “active bias”. We would like to note that an actual sudden explicit learning of the rule might have been overshadowed by an unclean execution of the rule by the participants. However, this is not very likely, as outside the reversal learning phase after a rule switch, the participants provide very stable responses, which raises the question of why they should only show unclean execution during reversal learning.

#### Rarely-rewarded experiment

##### Exploration bias is not learned

Surprisingly, when the never-rewarded positions become rarely rewarded, even though each is rewarded four times less often as each frequently rewarded position, no measurable exploration bias develops in human participants (Figure 4). In all three periods, the frequencies for rarely rewarded and frequently rewarded responses did not differ significantly (start: ${\tilde{\chi }}^{2}$ (1) = 1.72, p = 0.190; mid: ${\tilde{\chi }}^{2}$ (1) = 0.53, p = 0.468; end: ${\tilde{\chi }}^{2}$ (1) < 0.01, p = 0.962, $\alpha_{corrected}$ = $.01\bar{6}$, Bonferroni corrected for three tests). Our model also shows no development of the exploration bias (start: ${\tilde{\chi }}^{2}$ (1) = 0.23, p = 0.633; mid: ${\tilde{\chi }}^{2}$ (1) = 0.24, p = 0.626; end: ${\tilde{\chi }}^{2}$ (1) = 1.21, p = 0.272, $\alpha_{corrected}$ = $.01\bar{6}$, Bonferroni corrected for three tests).

##### Unrewarded exploration trials do not decrease

Since no exploration bias develops in the rarely-rewarded experiment, there is also no reduction in the number of unrewarded exploration trials. For both the model (*M* = −0.06, SD = 0.34) and the participants (*M* = −0.09, SD = 0.23), the average obtained learning rate is not significantly larger than zero (one-tailed t-test for one sample; model: *t*(59) = −1.38, p = 0.914, *d* = −0.18, 95% CI -0.15 to 0.03; participants: *t*(9) = −1.1, p = 0.850, *d* = −0.37, 95% CI -0.25 to 0.08). For an ideal observer, a reduction in average unrewarded exploration trials from 1.5 (considering all responses equally during exploration) to 0.86 (preferring the frequently rewarded responses) would be expected (see method details for more details).

### Model explanation

#### Fixed STN → GPe connections prevent the bias

To better reveal the role of plasticity in the STN → GPe projection, we ran 60 simulations for the never-rewarded experiment with a reduced model containing a fixed STN → GPe projection. If this reduced model does not learn the exploration bias, we can rule out that this behavior originates from learning in the cortico-striatal or cortico-subthalamic projections, since these projections remain plastic in the reduced model. Our simulation results show that the reduced model does not learn the exploration bias (see Figure S6). No significant differences were observed in the selection frequency between the never-rewarded and frequently rewarded positions (start: ${\tilde{\chi }}^{2}$ (1) = 0.44, p = 0.508; mid: ${\tilde{\chi }}^{2}$ (1) = 0.26, p = 0.611; end: ${\tilde{\chi }}^{2}$ (1) = 0.01, p = 0.908, $\alpha_{corrected}$ = $.01\bar{6}$, Bonferroni corrected for three tests). As a result, for the model with fixed STN → GPe projections the number of unrewarded exploration trials is not significantly reduced in the course of the task. The average obtained learning rate is not significantly larger than zero (*M* = −0.16, SD = 0.29, one-tailed t-test for one sample: *t*(59) = −4.14, *p* > 0.999, *d* = −0.54, 95% CI -0.23 to −0.08). Accordingly, the learning rate of the fixed model is significantly smaller than the learning rate of the plastic model and the learning rate of the participants (two-tailed t-test for independent samples: fixed vs. plastic: *t*(118) = 11.93, *p* < 0.001, $d_{z}$ = 2.2, 95% CI 0.54 to 0.76; fixed vs. participants: *t*(68) = 5.76, *p* < 0.001, $d_{z}$ = 2.0, 95% CI 0.4 to 0.82). There is even a small increase in the number of unrewarded exploration trials in the fixed STN → GPe model, since the simple deactivation of a model component, without further parameter adjustments, typically leads to a deterioration of the model performance.

##### A cluster connectivity pattern develops in STN → GPe

**Figure 6:**
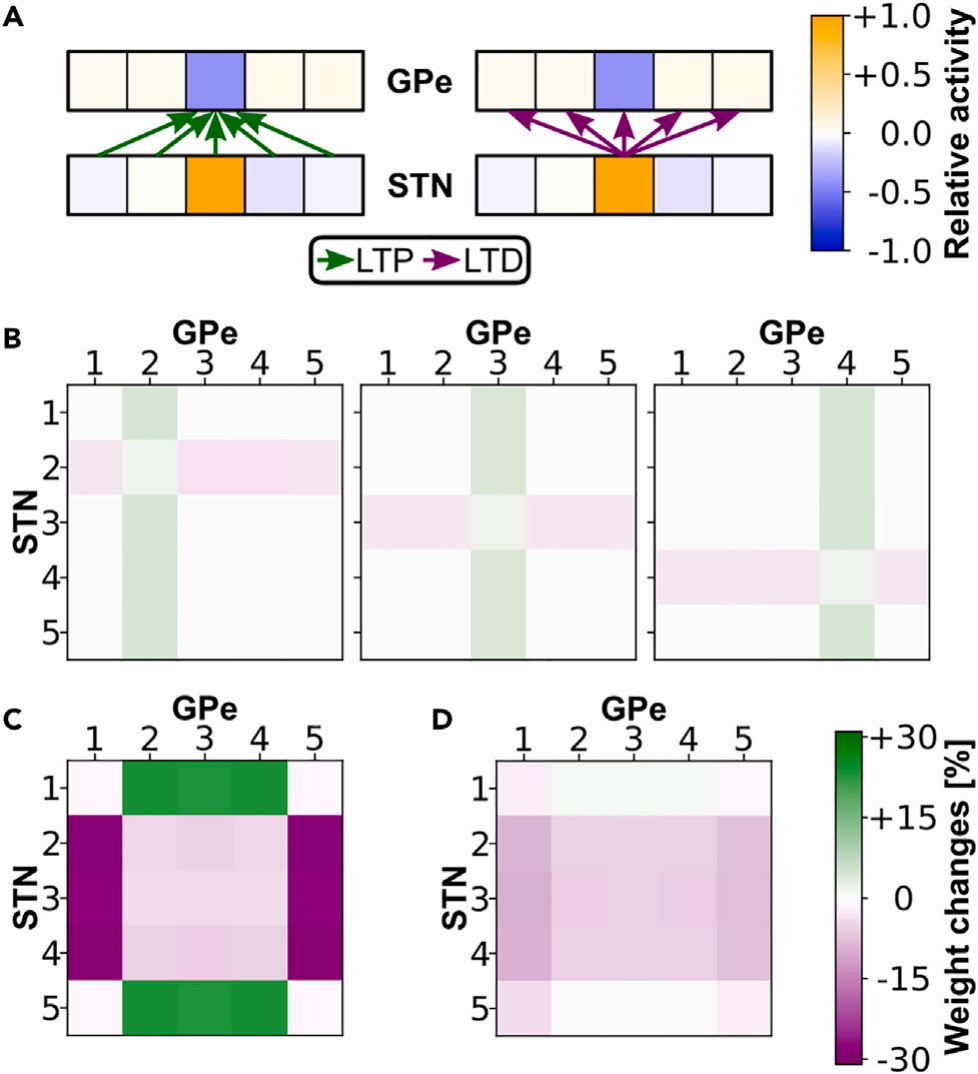
Activation profile in the STN-GPe circuit and resulting learned connectivity pattern in the STN → GPe projection (A) Relative activity in the STN-GPe sub-populations after a rewarded response, here selecting ”position 3” (averaged over 15 consecutive rewarded trials of one block). The color of each box represents the relative deviation of the average firing rate of the neurons in the 300 ms period after the dopamine signal increase relative to the firing rate of the entire trial ($\Delta \bar{r}=\left({\bar{r}}_{300}-{\bar{r}}_{total}\right)/{\bar{r}}_{total}$). Due to the homeostatic learning rule the weights from the neurons of all the STN channels toward the GPe neurons of the selected sub-channel increase (LTP) and the weights from the neurons of the selected STN sub-channel to neurons of all GPe channels decrease (LTD), after a phasic increase in the dopamine level. (B) Average weight changes between the neurons from the STN to the GPe sub-channels obtained in trials of 3 different rewarded positions (summed over 10 consecutive rewarded trials for each response). The plot on the left shows the average weight changes when response 2 was selected, the center plot when response 3 was selected and the right plot when response 4 was selected. (C) Weight changes across all blocks of the never-rewarded experiment (where only responses 2, 3, and 4 were rewarded) averaged over all 60 simulations. According to the resulting cluster connectivity pattern, neurons of the STN sub-channels project more strongly to those GPe sub-channels that are linked to the frequently rewarded responses, particularly because those to the sub-channels linked to the never-rewarded responses are declined. (D) Weight changes across all blocks of the rarely-rewarded experiment (where responses 2, 3 and 4 were frequently rewarded, and responses 1 and 5 rarely rewarded) averaged overall 60 simulations. The weights from the STN to the sub-channels linked to the rarely rewarded responses decreased, but much less than in the never-rewarded experiment. The weight changes across all blocks of the never- and rarely-rewarded experiment of the original model are shown in Figure S7. STN – subthalamic nucleus, GPe – external globus pallidus, LTP – long-term potentiation, LTD – long-term depression.

Besides the model responses, we analyzed the activity and connectivity patterns in the STN-GPe circuit in the fully learning model (Figure 6). Changes in the synaptic weights of the STN → GPe projection are defined by our new dopamine-modulated learning rule whose components originate from homeostatic plasticity.^59^^,^^60^ During positive-reward prediction errors, long-term potentiation (LTP) is caused by below-average neuronal activity (pre- or post-synaptical), and long-term depression (LTD) is caused by above-average activity.

Following each selection, SC-feedback toward the striatum increases the firing rate of the StrD1 and StrD2 sub-population encoding the saccade to the selected position. The StrD2 population inhibits GPe and disinhibits the STN neurons of the same sub-channel (Figure 6A). During consecutive rewarded trials, these activity patterns are repeated causing LTP in the synapses from all STN neurons to those GPe neurons of the selected sub-channel and LTD in the synapses of the neurons from the selected STN sub-channel to all GPe neurons, as shown in Figure 6A. The resulting weight changes of both LTD and LTP across multiple trials lead to a decline of connections to unrewarded sub-channels and an increase to rewarded sub-channels (Figure 6B). Since three positions are frequently rewarded in the never-rewarded experiment, a cluster connectivity pattern develops in the STN → GPe projection weights during the course of learning (Figure 6C). Either weights increase from STN to the GPe sub-populations encoding the frequently rewarded positions (rows 1 and 5 in Figure 6C), or weights decrease from STN to the GPe sub-populations encoding the never-rewarded positions (rows 2, 3, and 4 in Figure 6C). Either way, after enough training, each STN sub-population has stronger connections to the GPe sub-populations encoding the frequently rewarded positions than to the GPe sub-populations encoding the never-rewarded positions. This means that when an STN sub-population becomes active during exploration, it excites the GPe sub-populations that encode the frequently rewarded positions more strongly, biasing selection toward those positions.

In the rarely-rewarded experiment, the connection strengths to the GPe sub-populations encoding the rarely rewarded positions also decrease but much less (Figure 6D). This is because the reward signal for selecting the frequently rewarded positions is on average lower than the reward signal for selecting the rarely rewarded positions, because the reward prediction signal for the frequently rewarded positions is higher (see method details). As learning in the STN → GPe projection is dopamine-modulated, the larger weight changes caused by selecting the rarely rewarded positions compensate for the more frequent but weaker weight changes caused by selecting the frequently rewarded positions. This prevents the formation of the cluster connectivity pattern as in the never-rewarded experiment.

##### The STN → GPe cluster connectivity pattern biases exploration behavior

**Figure 7:**
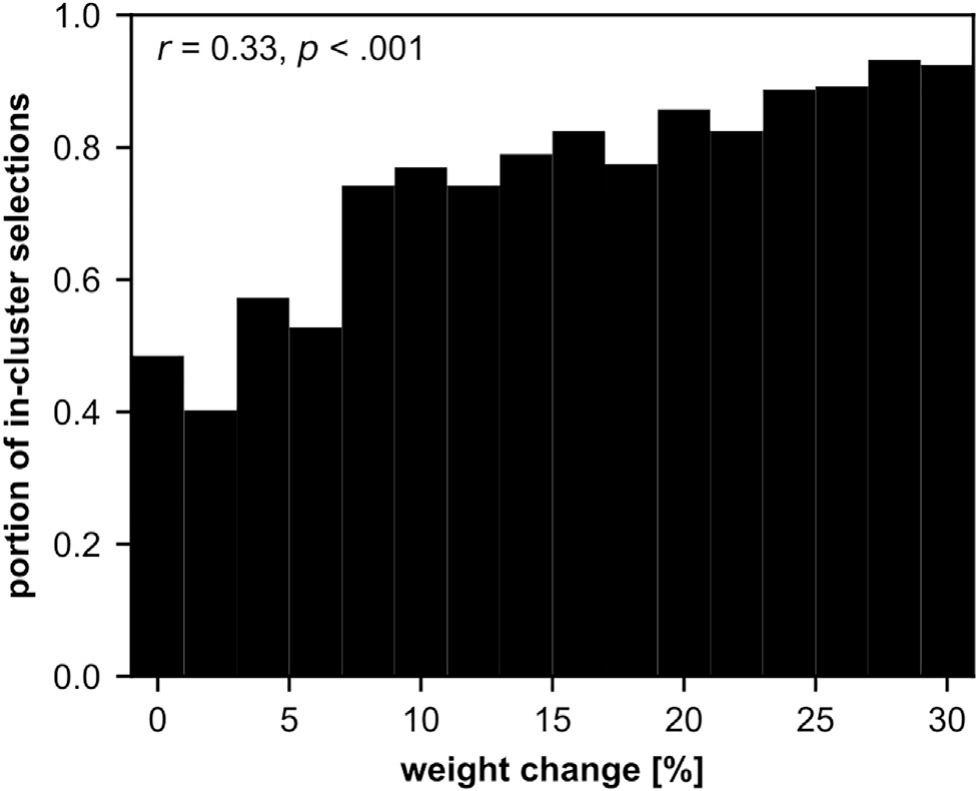
The dependence of initial exploration selections on the STN → GPe weight changes We simulated 6400 individual exploration phases, gradually varying STN → GPe connectivity (every 400 exploration phases, in 2% increments) from a uniform distribution toward the cluster connectivity pattern that emerges in the never-rewarded experiment. Each bar shows the proportion of 400 exploration phases with equal STN → GPe connectivity in which the first exploration selection represents a selection of a frequently rewarded response. This proportion increases with increasing weight change (reflecting the manifestation of the cluster connectivity pattern). The Spearman correlation between weight change and “first exploration selection is frequently rewarded” (either 0 - not frequently rewarded or 1 - frequently rewarded) is shown in the upper left corner. STN – subthalamic nucleus, GPe – external globus pallidus.

We investigated the relationship between the formation of the cluster connectivity pattern in the STN → GPe projection and the model’s exploration behavior by performing further simulations focusing on the exploration phase. For this purpose, we initialized the synaptic weights of the plastic cortical projections as they would be after reversal and simulated a single exploration phase. Importantly, we varied the initial synaptic weights of the STN → GPe plastic connections for different simulations so that they were either more similar to the initial weights (uniform distribution) or more similar to the resulting cluster connectivity pattern from the never-rewarded experiment (see Figure 6C). A total of 6,400 simulations were performed in which weight changes were increased in 2% increments from 0% to +30% (yielding 16 different initial synaptic weights for STN → GPe with 400 simulations each, see Figure 7). The more the weights approximated the cluster connectivity pattern, the higher the probability that the first explored response during an exploration phase originated from the cluster (Spearman correlation, *r*(6398) = 0.33, p< 0.001, 95% CI 0.31 to 0.36). This explains why the cluster connectivity pattern in the never-rewarded experiment causes the exploration bias, while the weight pattern resulting from learning in the rarely-rewarded experiment is not sufficient.

## Discussion

We designed a novel 5-choice reversal learning task with alternating position-reward contingencies to study exploration behavior in human participants after a reversal, different from previous reversal learning experiments,^7^^,^^8^^,^^14^^,^^24^^,^^61^^,^^62^ which did not study exploration behavior. Particularly, we were interested in the question if exploration behavior depends on previous experience, that is, if previously rewarded responses are more often explored than responses that have been not rewarded and how quickly such a beneficial bias is established.

The motivation behind our experimental investigation arose from our modeling studies of the basal ganglia. Predictions obtained from our neuro-computational model suggest that exploration behavior can be guided by the STN-GPe circuit. Responses, frequently rewarded in the past, are prioritized during exploration after a rule change.

This model prediction is different from previous experimental and modeling studies which demonstrated that the indirect and hyperdirect pathways of the basal ganglia solely actively suppress irrelevant or unwanted actions.^32^^,^^38^^,^^41^^,^^63^ However, it has been shown that typically concurrent activation of striatal spiny projection neurons (SPNs) of the direct as well as indirect pathway contribute to the final selection.^64^ These pathways can simultaneously favor the selection of wanted and the suppression of unwanted actions to determine the final response but cannot directly bias exploration behavior. According to our model, a few D2-type cells of the indirect pathway selectively increase their firing rate when unrewarded responses occur (negative reward prediction error), which in turn suppresses the particular action that has led to the unrewarded responses. Immediately after a change in reward contingency, the previously rewarded action is still favored by the direct pathway but becomes increasingly blocked by the indirect pathway. However, the activation of the indirect pathway not only travels from D2-type cells via GPe to SNr and suppresses the SC, but our model predicts that the activation also travels via GPe to STN and activates now different GPe cells which now disinhibit parts of the SC, and thus induce an exploration bias. This model prediction matches well the observation of switch selection during optogenetic activation of the indirect pathway.^65^ As the memory stored in the STN-GPe circuit is only recalled upon the activation of specific StrD2 cells after negative reward prediction errors, it does not interfere with normal selection behavior. Thus, the exploration bias is selectively switched on when required and is silent otherwise.

Somewhat surprisingly, we did not observe an exploration bias, when we change our setting toward rarely rewarded positions as an ideal observer would account for the frequency of rewards and predict a bias. According to our model, the dopaminergic reward prediction error can explain this effect, because rarely rewarded positions, when visited and rewarded, lead to a larger prediction error and thus, induce larger neural weight changes than at those positions that are more often rewarded.

Despite our observation that human exploration behavior matches our model prediction very well and although the basal ganglia is one of the key structures in guiding saccades to rewarded positions,^43^^,^^54^^,^^55^^,^^56^^,^^66^ we cannot rule out the involvement of other brain areas by means of a model. Nevertheless, our prediction has several merits. The STN-GPe circuit is simple and provides an elegant solution for focusing exploration to promising candidates. A putative cortical circuit will likely be more complex and—if explicit-rule-based—would require maintaining reward statistics for each saccade target. Further, we have shown that the learned exploration bias develops continuously and does not suddenly become active, arguing against an explicit rule-based behavior involving higher cortical areas. Finally, we investigated the human behavior with oculomotor responses and encouraged fast responses, which—at least to some degree—reduce the influence of cognitive planning and deliberation.

In summary, our results indicate a basal ganglia contribution to the observed exploration bias in human saccadic selection behavior after a change in position-reward contingency. Specifically, our model predicts a new function of the STN-GPe circuit that can learn to selectively stimulate alternative promising choices. This provides an advantage over randomly exploring all possible responses.

### Limitations of the study

Our study demonstrates that a model of the basal ganglia predicts human behavior in a 5-choice reversal learning task. However, we would refrain from concluding that the observed human behavior is fully determined by the basal ganglia. Our model suggests a plausible and simple implementation in a subcortical circuit which can explain the observed behavior, but as with any neuro-computational modeling study, we cannot rule out other possible implementations in the brain. The disengagement from previously rewarded responses is faster in humans and may likely be dominated by rule-based reasoning^5^^,^^67^ rather than fast but still gradual reinforcement learning in the basal ganglia. The exploration behavior has been explained as a result of learning in the STN-GPe circuit, but our study cannot rule out that a similar behavior could originate in the parietal cortex, the supplementary eye fields, or the dorsolateral prefrontal cortex.

## STAR★Methods

### Key resources table

**Table:** 

REAGENT or RESOURCE	SOURCE	IDENTIFIER
**Deposited data**		

Human and simulation data generated in the study	Zenodo	[Zenodod Data]: [https://doi.org/10.5281/zenodo.6546571]

**Software and algorithms**		

Code for performing the simulations (with the model) and for analyzing the generated data (human and simulated)	Zenodo	https://doi.org/10.5281/zenodo.6555886
scipy v1.7.1, Python module	Virtanen et al.^79^	https://doi.org/10.1038/s41592-019-0686-2
pingouin v0.5.1, Python module	Vallat et al.^80^	https://doi.org/10.21105/joss.01026
ANNarchy v4.7.1.4, Python module	Vitay et al.^72^	https://doi.org/10.3389/fninf.2015.00019
Psychophysics Toolbox for Matlab	Brainard et al.^76^	https://doi.org/10.1163/156856897X00357
EyeLink Toolbox for Matlab	Cornelissen et al.^78^	https://doi.org/10.3758/BF03195489

### Resource availability

#### Lead contact

Further information and requests for resources should be directed to and will be fulfilled by the lead contact, Fred Hamker (fred.hamker@informatik.tu-chemnitz.de).

#### Materials availability

No other materials besides data and code were generated in this study.

#### Data and code availability

• The human and simulation data generated in this study have been deposited at Zenodo and are publicly available as of the date of publication. DOIs are listed in the key resources table.
• All original code has been deposited at Zenodo and is publicly available as of the date of publication. DOIs are listed in the key resources table.
• Any additional information required to reanalyze the data reported in this paper is available from the lead contact upon request.

### Experimental model and subject details

Twenty students (never-rewarded experiment: 8 females, 2 males; mean age = 20.5, SD = 2.9, rarely rewarded experiment: 5 females, 5 males; mean age = 26.2, SD = 5.1) participated in the experiments and gave written informed consent to participate. No participant took part in both versions of the experiment. All had normal or corrected-to-normal vision and normal color vision. All procedures were evaluated by the responsible local ethics board (Ethikkommission HSW, Chemnitz University of Technology) who decided that no in-depth assessment is required (case no.: V-318-PHKP-WET-Basalgang-04032019). All participants were naive with respect to the details of the reward contingency.

### Method details

#### Model details

The individual neuron populations shown in Figure 2, except for the cortex, each consist of 500 single-compartment Izhikevich spiking neurons.^68^ The striatum is separated in two groups: D1 dopamine receptor expressing cells (direct pathway) and D2 dopamine receptor expressing cells (indirect pathway). Each population is further divided in five sub-populations (each with 100 neurons), one for each of the five possible saccade target position. The membrane potential of each cell is given by:

(Equation 1) $$\begin{array}{l} C\frac{dV}{dt}=n_{2}V^{2}+n_{1}V+n_{0}-U+I\\ \frac{dU}{dt}=a\left(b\left(V-v_{r}\right)-U\right)\\ \text{if}\;V\geq v_{peak}\;\text{then}\left\{ \begin{array}{ccc} V & \leftarrow & c\\ u & \leftarrow & u+d \end{array}\right. \end{array}$$

where *V* is the membrane potential, *U* is the recovery variable and *I* is the synaptic current. The parameters depend on the neuron’s population and are shown in Table S1. Both striatal populations use the parameters proposed by Humphries, Wood, and Gurney.^69^ The SNr, GPe and STN parameters are from Thibeault and Srinivasa.^70^ The SC is modeled with 5 discrete positions encoded by its 5 sub-populations as saccade targets and not as a continuous space model. Its neuron parameters are identical to the ones of the thalamo-cortical spiking model proposed by Izhikevich^68^ to allow rebound activity after disinhibition. The SC includes inhibitory interneurons for which we used the fast spiking model of Izhikevich.^68^

The synaptic current *I* in each cell is described by the following equation:

(Equation 2) $$\begin{array}{l} I=I_{base}-g_{AMPA}\left(V-E_{AMPA}\right)-g_{GABA}\left(V-E_{GABA}\right)\\ \tau \frac{dg_{AMPA}}{dt}=-g_{AMPA}\\ \tau \frac{dg_{GABA}}{dt}=-g_{GABA} \end{array}$$

When an action potential arrives at the synapse, we instantaneously increase the conductance (for excitatory connections: $g_{AMPA}$ and for inhibitory connections: $g_{GABA}$) on the postsynaptic cell by the weight value of the synaptic connection, otherwise both conductances decay exponentially with time constant τ=80 ms. Conductances larger than zero cause synaptic currents which drive the membrane potential toward their corresponding reversal potential of $E_{AMPA}=0$ mV and $E_{GABA}=-90$ mV. $I_{base}$ refers to the constant baseline current in the absence of other synaptic current, which regulates the level of spontaneous activity of neurons.

Independent neuronal noise (see Table S2) is induced in the SNr, GPe, SC and STN, for each neuron by a spiking excitatory input following a Poisson distribution (referred to as Poisson neuron). In striatal cells the noisy input consists of excitatory and inhibitory spiking input to keep them silent without further excitatory input.

Cortical cells are represented by a population of 100 Poisson neurons which activate with a fixed average firing rate if the stimulus is present and are silent otherwise. All cortical cells are initially connected to all striatal and subthalamic cells and follow a dopamine-modulated spike time dependent plasticity rule,^44^^,^^58^ motivated by physiological observations^57^:

(Equation 3) $$\begin{array}{l} \tau_{pre}\frac{dA_{pre}}{dt}=-A_{pre}+\Delta_{pre}X_{pre}\left(t\right)\\ \tau_{post}\frac{dA_{post}}{dt}=-A_{post}+\Delta_{post}X_{post}\left(t\right)\\ \tau_{E}\frac{dE}{dt}=-E-X_{pre}\left(t\right)A_{post}+X_{post}\left(t\right)A_{pre}\\ \frac{dw}{dt}=\alpha \cdot E\cdot DA\left(t\right)-\delta \cdot X_{pre}\left(t\right) \end{array}$$

Where $A_{pre}$ and $A_{post}$ are spike traces for the presynaptic and postsynaptic cell respectively. Both increase by a factor $\Delta_{pre}$ or $\Delta_{post}$, each time the corresponding cell fires and decay exponentially with time constant $\tau_{pre}$ or $\tau_{post}$. $X_{pre}\left(t\right)$ and $X_{post}\left(t\right)$ are 1 if the corresponding cell has fired at time *t* and 0 otherwise. *E* is an elegibility trace, which is increased with each pre-post spike pair and decreased with each post-pre spike pair. The weight change is finally computed by multiplying *E* with a learning rate, α, and with a reward prediction error signal,^71^ DA (t). DA (t) is positive if dopamine is above baseline (positive reward prediction error), negative if below (negative reward prediction error) and 0 otherwise. Increases in the level of dopamine during trials in which the rewarded response is selected lead to long-term potentiation (LTP) in the direct and hyperdirect pathway and long-term depression (LTD) in the indirect pathway. Unrewarded responses lead to a negative DA (t) and thus to the contrary effects. A normalization term ($\delta \cdot X_{pre}\left(t\right)$) slightly reduces the weight after every presynaptic spike. All parameters for the learning rules of the plastic cortical projections are presented in Table S3.

The model includes plastic synapses between the cortex and the SC to mimic a cortical activation pathway to the SC. These connections are not modulated by dopamine and thus follow a more simple spike time depend learning rule:

(Equation 4) $$\begin{array}{l} \tau_{pre}\frac{dA_{pre}}{dt}=-A_{pre}+\Delta_{pre}X_{pre}\left(t\right)\\ \tau_{post}\frac{dA_{post}}{dt}=-A_{post}+\Delta_{post}X_{post}\left(t\right)\\ \frac{dw}{dt}=-X_{pre}\left(t\right)A_{post}\left(t\right)+X_{post}A_{pre}\left(t\right)-\delta \cdot X_{pre}\left(t\right) \end{array}$$

The connectivity patterns of all fixed projections are defined stochastically, according to the probabilities shown in Table S4.

Finally, we defined one linear integrator for each saccade target position. These units increase their value after each SC spike emitted by a cell in their corresponding sub-channel. Under absence of activity they decrease exponentially to 0. The response whose associated integrator reaches a fixed threshold first or is largest after a maximum stimulus presentation time is considered the selected response.

The model is implemented in the neurosimulator ANNarchy ver. 4.7.1.4.^72^ All time-dependent variables are updated each time step dt = 0.1 ms. Differential equations are solved with the forward Euler method.

#### Simulations - procedure details

All simulations were performed with different random number generator seeds, thus, different initial weights in the plastic Cortex → StrD1 projection, different sequences of rewarded positions, and different independent neuronal noise. We simulated three different versions of our 5-choice reversal learning task - the never-rewarded, rarely rewarded, and all-rewarded experiment. The never-rewarded experiment simulations consist of 60 blocks in which 3 out of 5 responses are frequently rewarded with equal probability. The rarely rewarded experiment simulations consist of 70 blocks. Within each 14 blocks, 3 out of 5 responses are rewarded 4 times each and the 2 remaining responses are rewarded only once. Thus, there are 3 frequently rewarded and 2 rarely rewarded responses evenly distributed over the entire experiment. The all-rewarded experiment simulations consist of 60 blocks in which all 5 responses are rewarded with equal probability. Blocks changed after 15 consecutive rewarded trials or a maximum of 30 trials. The same response was never rewarded in two consecutive blocks.

During every trial of the simulation, a resting phase of 1.7 s was simulated first. After that, the stimulus (cortex) was activated and the model had to respond within 1 s. After the model’s response or after the maximum stimulus presentation time of 1 s, the stimulus was deactivated. Then, after a 130 ms delay, the reward signal was given to the model. After that, the next trial started again with a resting period.

As mentioned in method details, there are multiple dopamine-modulated plastic projections. During reward delivery (130 ms after stimulus offset), the dopamine signal DA of the projections is set to the reward signal *R* and then decays exponentially with the time constant $\tau_{DA}$, which depends on the corresponding projection (Equation 5, leaving only a small window of time for dopamine-modulated plasticity. The reward signal *R* depends on the response of the model and is calculated from a reward constant r=0.25 and a response-specific reward prediction signal $P_{i}$. The reward prediction signal $P_{i}$ (Equation 7 is updated at the occurrence of the corresponding response *i* using the time constant $\tau_{P}=50$. For the original model simulations we used a different response-unspecific reward prediction signal *P* (Equation 8 which is increased with each rewarded response using the time constant $\tau_{P}=60$ and reset to zero with each unrewarded response.

(Equation 5) $$\begin{array}{l} DA\leftarrow R,\text{reward}\;\text{delivery}\\ \tau_{DA}\frac{dDA}{dt}=-DA \end{array}$$

(Equation 6) $$R=\left\{ \begin{array}{cc} r-P_{i}\text{,} & \text{rewarded}\;\text{response}\;i\\ -r\text{,} & \text{unrewarded}\;\text{response}\\ -r/2\text{,} & \text{missing}\;\text{response} \end{array}\right.$$

(Equation 7) $$P_{i}=P_{i}+\frac{r}{\tau_{P}}\cdot \left\{ \begin{array}{cc} 1\text{,} & \text{rewarded}\;\text{response}\;i\\ -1\text{,} & \text{unrewarded}\;\text{response}\;i \end{array}\right.$$

$$-r\leq P_{i}\leq 0.8r$$

(Equation 8) $$P=\left\{ \begin{array}{cc} P+\frac{r-P}{\tau_{P}}\text{,} & \text{rewarded}\;\text{response}\\ 0\text{,} & \text{unrewarded}\;\text{response} \end{array}\right.$$

#### STN-GPe learning rule

The newly introduced learning rule of the STN → GPe projection is based on homeostatic plasticity.^59^^,^^60^ It has an LTP component caused by below-average activity and an LTD component caused by above-average activity.

(Equation 9) $$\begin{array}{l} \tau_{A}\frac{dA_{pre}}{dt}=-A_{pre}+\Delta A\cdot X_{pre}\left(t\right)\\ \tau_{A}\frac{dA_{post}}{dt}=-A_{post}+\Delta A\cdot X_{post}\left(t\right)\\ \tau_{\bar{A}}\frac{d{\bar{A}}_{pre}}{dt}=A_{pre}-{\bar{A}}_{pre}\\ \tau_{\bar{A}}\frac{d{\bar{A}}_{post}}{dt}=A_{post}-{\bar{A}}_{post}\\ \Delta_{LTP}={\left(0.9-\frac{A_{pre}}{{\bar{A}}_{pre}}\right)}^{+}+\epsilon \cdot {\left(0.9-\frac{A_{post}}{{\bar{A}}_{post}}\right)}^{+}\\ \Delta_{LTD}={\left(\frac{A_{pre}}{{\bar{A}}_{pre}}-1.1\right)}^{+}+\epsilon \cdot {\left(\frac{A_{post}}{{\bar{A}}_{post}}-1.1\right)}^{+}\\ \tau_{w}\frac{dw}{dt}={\left(DA\left(t\right)\right)}^{+}\cdot \left(\left(1-\frac{w}{w_{\max}}\right)\cdot \Delta_{LTP}-\left(\frac{w}{w_{\max}}\right)\cdot \Delta_{LTD}\right) \end{array}$$

$A_{pre}$ and $A_{post}$ are spike traces of the corresponding neurons. ${\bar{A}}_{pre}$ and ${\bar{A}}_{post}$ are the temporal mean of these traces. The ratio between *A* and $\bar{A}$ is used to determine phasic changes in activity. Here, a threshold of 10% is used to exclude noise and small changes due to the shape of the spike traces (continuous increase and decay in a small range). Thus, a phasic increase is detected when $A/\bar{A}>1.1$ and a phasic decrease when $A/\bar{A}<0.9$. The LTP is active in case of a phasic decrease in activity and LTD is active in case of a phasic increase in activity. Otherwise, both are zero. The final weight change is computed as the sum of the (positive) LTP and (negative) LTD. Each component is scaled based on the ratio between the current weight and the maximum weight. Thus, the effect of LTP is stronger at lower weights and converges to zero at larger weights, and inversely for LTD. The weights therefore self-regulate. As the resulting weighted sum is multiplied by the positive part of the dopamine signal, only dopamine increases lead to weight changes. The parameters $\tau_{A}$, $\tau_{\bar{A}}$ and $\tau_{w}$ are time constants. The parameter ΔA is the amount a trace is increased after a spike. The parameter ε ensures that activity changes of the postsynaptic neuron have a stronger influence on the weight changes than that of the presynaptic neuron. The parameters are given in Table 1.

Many studies have already observed plasticity in the STN-GPe circuit,^45^^,^^46^^,^^47^^,^^48^^,^^49^^,^^50^^,^^51^ but most studies only refer to the GPe → STN projection. Hanson and Jaeger^45^ found that plasticity in the STN → GPe projection is best described by separate potentiation and depression elements, similar to our learning rule. Further, dopamine innervates the STN and GPe and the contribution of dopamine in plasticity is well known.^48^^,^^52^^,^^53^^,^^57^^,^^73^^,^^74^^,^^75^ However, our learning rule is set to implement a particular function based on plausible components. Due to a lack of detailed studies, data is still not conclusive to derive a learning rule bottom-up on data only.

#### Eye-tracking – Setup and stimuli

Stimuli were generated using MATLAB (MathWorks, Natick, MA) and the Psychophysics Toolbox.^76^^,^^77^ Stimuli were presented at a viewing distance of 57 cm on a 523 × 300 mm large ViewPixx3D Full Monitor (VPixx Technologies) with a spatial resolution of 1920 × 1080 pixels and a refresh rate of 120 Hz. Eye movements were recorded with an Eyelink-1000 Plus device (SR Research, Ottawa, ON, Canada) in tower-mount configuration using the EyeLink Toolbox.^78^ Only the left eye was tracked.

Stimuli were presented on a gray background. The initial fixation cross was presented in black color in the center of the screen with a width and height of 0.5 degrees of visual angle (dva). The 5 possible saccade target positions were given by white circles with a radius of 0.25 dva arranged in a circle equally spaced around the center of the screen (radius = 10 dva). The selection of a position was determined in two steps. First, gaze is required reach the target area, which means it has to exceed a circle around the center of the screen (radius = 7.5 dva) and second the position closest to gaze was selected.

#### Eye-tracking – Procedure details

Prior to the experiments, participants were informed about the general procedure and the meaning of the different reward-feedback colors. They were instructed to avoid negative reward feedback (red for wrong choices and blue for too slow responses) and informed that on each trial only one saccade target is rewarded and that position-reward contingencies will change during the experiment.

The general procedure of the experiments and the timings of the trials are shown in Figure 1. During the trials of the experiment, participants first had to fixate a central fixation cross for at least 1 s. After the fixation was correctly detected, the fixation cross disappeared and the stimulus, consisting of 5 white circles at different positions, was presented until the participant’s response was detected or for a maximum of 0.4 s. After the participant’s response, the reward feedback was presented for 0.4 s. If no response occurred during the 0.4 s stimulus presentation the reward feedback indicated that the response was too slow. After the presentation of the reward feedback, the next trial started again with the fixation cross.

Both versions of the experiment were divided into six parts separated by resting breaks of several minutes. In the never-rewarded experiment, the first two parts were used to familiarize the participants with the task, to avoid any bias due to training. These two parts consisted of 12 blocks each, resulting in 24 blocks in which all five possible responses were (almost) equally frequently rewarded (four responses rewarded in five blocks each, a single random response rewarded in four blocks). In the rarely rewarded experiment, only the first part consisting of 15 blocks (all five responses rewarded in three blocks each) was used for the familiarization phase.

In the never-rewarded experiment, the remaining four parts after the familiarization phase consisted of 12 blocks resulting in 48 blocks in total in which three responses were frequently rewarded and two responses were never rewarded (in each part: each frequently rewarded response rewarded in four blocks, resulting in 12 blocks per part). In the rarely rewarded experiment, the remaining five parts after the familiarization phase consisted of 14 blocks resulting in 70 blocks in total in which three responses were frequently rewarded and two responses were rarely rewarded (in each part: each frequently rewarded response rewarded in four blocks and each rarely rewarded response rewarded in one block, resulting in 14 blocks per part). The three frequently rewarded positions for each participant were determined prior to the experiment, such that each subset of positions was used in exactly one participant. This also determined the number of participants as the minimum number for which such counterbalancing was possible to 10 (= 5 choose 3) for each version of the experiment.

In both experiments, the rewarded position changed between the blocks. The blocks changed after 7–9 consecutive rewarded trials or a maximum of 12–14 trials. These values were drawn randomly from a uniform distribution for each block, to prevent counting trials and anticipating the reversals.

#### Ideal observer details

To evaluate the number of unrewarded exploration trials of the participants and the model, we compared them to the number of unrewarded exploration trials of a fictitious ideal observer. For this observer, we assume the following: The observer excludes unrewarded responses. Without exploration bias, the observer chooses all possible responses (that the observer does not exclude) with equal probability. With exploration bias, the observer first chooses all responses that are within the bias with equal probability and excludes all responses outside the bias. Once all responses inside the bias have been chosen and not rewarded, the observer chooses all responses outside the bias with equal probability. Once a response is rewarded, the exploration is over.

Using these rules, we have described the selection behavior during exploration of the ideal observer with and without exploration bias for the different versions of the experiment as decision trees. Such a decision tree shows all possible decision paths of the observer and their probabilities during exploration. From the probabilities of the individual decision paths and the number of unrewarded exploration trials included, the expected value for the unrewarded exploration trials can be determined. An example of such a decision tree is shown in Figure S8. We obtained the following expected values: never-rewarded experiment with bias: 0.50; never-rewarded experiment without bias: 1.50; rarely rewarded experiment with bias: 0.86; rarely rewarded experiment without bias: 1.50.

### Quantification and statistical analysis

#### Analyzed data

For all behavioral analyses, only successfully completed blocks were analyzed. Any block containing 7 consecutive rewarded trials was considered successful. The trials of each analyzed block were divided into consecutive unrewarded selections of the previously rewarded response, selections of other responses (referred to as exploration trials), and the consecutive rewarded selections of the newly rewarded response. The first selection of the consecutive rewarded selections of the newly rewarded response and sporadic selections of the previously rewarded response (after other responses were explored) are considered as exploration trials.

The data of the familiarization phases were not included in the evaluation. Further, the blocks, i.e. exploration phases, that followed the breaks were not included in the analysis.

#### Average selection frequencies of unrewarded exploration trials

To obtain the average selection frequencies of never/rarely rewarded and frequently rewarded positions, the selections during the exploration phases were divided into these two categories and counted. The counts were weighted as a function of the available unrewarded exploration selections per category during the corresponding exploration phase, since the available unrewarded exploration selections depend on the previously and newly rewarded positions (they do not count as unrewarded exploration selections).

In the never-rewarded experiment, there are always two available never-rewarded unrewarded exploration selections and only one available frequently rewarded one (because the previously and the newly rewarded positions are always two frequently rewarded positions). Therefore, the number of never-rewarded selections in this experiment is weighted by ½.

In the rarely rewarded experiment, there can be different pairs of previously and newly rewarded position (frequently rewarded - frequently rewarded, frequently rewarded - rarely rewarded, rarely rewarded - frequently rewarded, rarely rewarded - rarely rewarded). Thus, different exploration periods have different weights for the counts of the selections of frequently and rarely rewarded positions.

After the weighted counts for each exploration period are obtained, they are averaged across the human participants and the model simulations. An average value of 1 then means that in that exploration phase, all participants/simulations made all available unrewarded exploration selections in the corresponding category.

#### Exponential fit for analyses of unrewarded exploration trials

To quantify how the number of unrewarded exploration trials changes over time, we fitted an exponential function (Equation 10, see Figure S9) with the data from each participant and model simulation to obtain a learning rate for each participant/model simulation. This was motivated by the fact that the number of unrewarded exploration trials in the never-rewarded experiment converged from about 1.5 to about 0.5 (see Figure 4), which also reflect the expected numbers for an ideal observer (method details). The exponential function was fitted to the number of unrewarded exploration trials ($y_{target}$) as a function of exploration phase (*x*). For this, the exploration phases were divided by the maximum number of exploration phases for the respective experiment (never-rewarded experiment = 59, rarely rewarded experiment = 69) for both the model simulations and the participants, and thus restricted to the range 0–1.

The exponential function is defined by three parameters: the starting value $y_{0}$ and the asymptotic value $y_{1}$, which are determined by the experiment (never-rewarded experiment: $y_{0}$ = 1.5, $y_{1}$ = 0.5; rarely rewarded experiment: $y_{0}$ = 1.5, $y_{1}$ = 0.86), and the time constant $\tau_{\exp}$, which we tune during optimization using the ”optimize.curve_fit” function from the Python module scipy (v1.7.1,^79^). A special feature of the exponential function used is that negative time constants are allowed. This makes it possible to obtain exponential functions through the optimization that increase symmetrically to the initial value $y_{0}$, which prevents a bias toward decreasing exponential functions.

Half-lives $t_{1/2}$ were determined from the fitted exponential functions, which we restricted to the range −1 to 1 (Equation 11). Finally, the half-lives were converted to a learning rate γ (Equation 12) ranging from −1 (fast increasing number of unrewarded exploration trials) to 1 (fast decreasing of unrewarded exploration trials).

(Equation 10) $$y_{y_{0},y_{1},\tau_{\exp}}\left(x\right)=\left\{ \begin{array}{ll} \left(y_{0}-y_{1}\right)e^{-\tau_{\exp}x}+y_{1}\text{,} & \tau_{\exp}>0\\ -\left(y_{0}-y_{1}\right)e^{\tau_{\exp}x}+2y_{0}-y_{1}\text{,} & \tau_{\exp}\leq 0 \end{array}\right.$$

(Equation 11) $$t_{1/2}=\frac{\ln\left(2\right)}{\tau_{\exp}}-1\leq t_{1/2}\leq 1$$

(Equation 12) $$\gamma =\left\{ \begin{array}{cc} -1-t_{1/2}\text{,} & t_{1/2}<0\\ 1-t_{1/2}\text{,} & t_{1/2}>0 \end{array}\right.$$

#### Tools for statistical analyses

All statistical tests performed and their details, including the number of participants and simulations, are described in the results. Unless otherwise noted, a significance level of α=.05 was used. The tools used to perform the tests are described in the following sentences.

All t-tests were performed with the Python module scipy (v1.7.1,^79^) (for dependent samples: function ”stats.ttest_rel()”; for independent samples: function ”stats.ttest_ind()”; for one sample: function ”stats.ttest_1samp()”). The homogeneity of the variances of two independent samples was tested using the ”homoscedasticity” function from the Python module pingouin (v0.5.1,^80^). In case of unequal variances, Welch’s t-test was applied. The effect sizes were calculated as standardized differences Cohen’s $d_{z}$ for dependent samples and classical Cohen’s d for one sample and independent samples tests.

The Spearman correlation and the one-way chi-square tests were also performed with the Python module scipy using the functions ”stats.spearmanr()” and ”stats.chisquare()”.
