# Supplementary material for: Exploration behavior after reversals is predicted by STN-GPe synaptic plasticity in a basal ganglia model
Source: iScience. 2023 Apr 11;26(5):106599. doi: 10.1016/j.isci.2023.106599 (PMC10214406; doi:10.1016/j.isci.2023.106599)
Supplement: Document S1. Figures S1–S9 and Tables S1–S4 [file mmc1.pdf]

iScience, Volume *26*

Supplemental information

Exploration behavior after reversals is predicted by STN-GPe synaptic plasticity in a basal ganglia model

Oliver Maith, Javier Baladron, Wolfgang Einhäuser, and Fred H. Hamker

S 1 Supplemental figures

S 1.1 Original model exploration behavior prediction

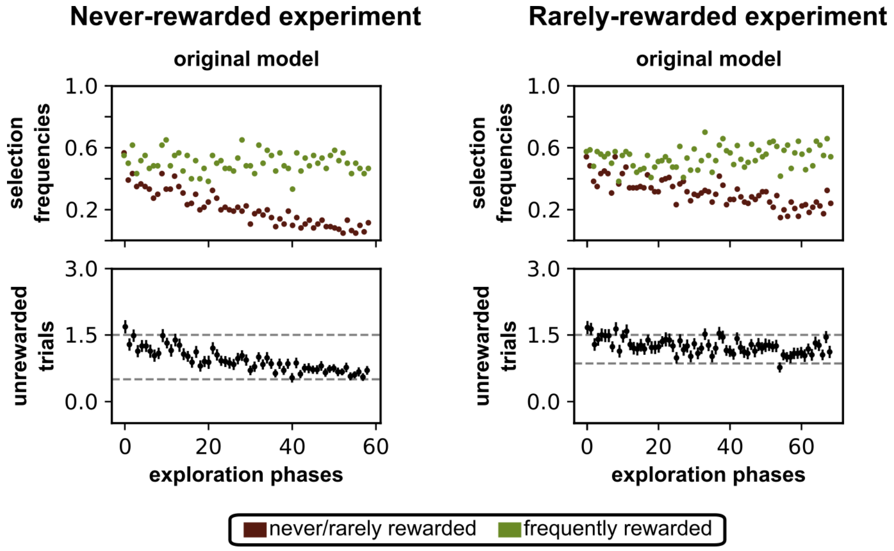

Figure S 1: Selection frequencies of unrewarded exploration trials of the original model for the never- and rarely-rewarded experiment, related to Figure 4. We simulated both versions of the experiment with two models, our original model and a revised model. The results of the revised model are shown in the main text and the results of the original model are shown here in the supplement. The data are presented as in Figure 4 of the main paper. For each experiment, the data are obtained from 60 simulations. An exploration bias is learned for both, the never-rewarded experiment (start: $\tilde{\chi}^2(1) = 0.01$, $p = .903$; mid: $\tilde{\chi}^2(1) = 5.65$, $p = .017$; end: $\tilde{\chi}^2(1) = 12.6$, $p < .001$, $\alpha_{corrected} = .01\overline{6}$, Bonferroni corrected for three tests) and the rarely-rewarded experiment (start: $\tilde{\chi}^2(1) = 0.06$, $p = .807$; mid: $\tilde{\chi}^2(1) = 3.77$, $p = .052$; end: $\tilde{\chi}^2(1) = 6.89$, $p = .009$, $\alpha_{corrected} = .01\overline{6}$, Bonferroni corrected for three tests). Also, in both experiments the learning rate is significantly larger than zero (never-rewarded: $M = 0.62$, $SD = 0.28$, one-tailed t-test for one sample: $t(59) = 17.08$, $p < .001$, $d = 2.22$, 95% CI 0.55 to 0.69; rarely-rewarded: $M = 0.39$, $SD = 0.34$, one-tailed t-test for one sample: $t(59) = 8.59$, $p < .001$, $d = 1.12$, 95% CI 0.30 to 0.47). Accordingly, in the rarely-rewarded experiment the learning rate of the original model is significantly larger than the learning rate of the participants (two-tailed Welch's t-test: $t(68) = 5.23$, $p < .001$, $d_z = 1.42$, 95% CI 0.25 to 0.70).

S 1.2 Original model global performance results

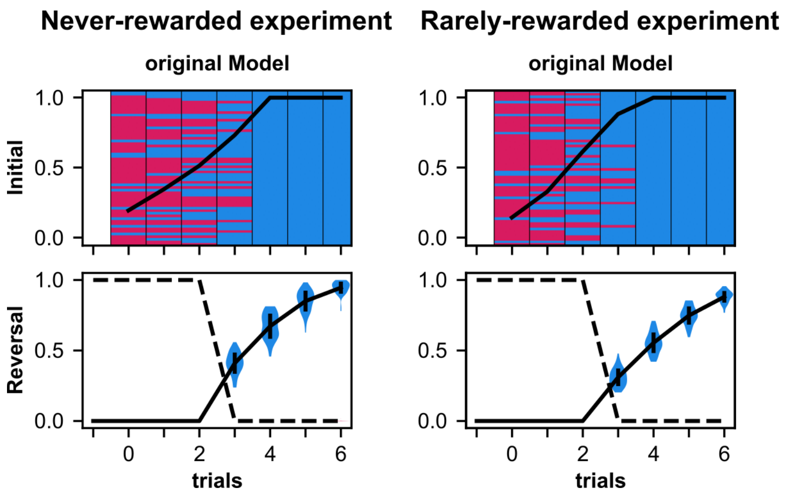

Figure S 2: Performance of the original model during the first seven trials of the initial learning phase and reversal learning phases for the never- and rarely-rewarded experiment, related to Figure 3. The data are presented as in Figure 3 of the main paper. For each experiment, data are obtained from 60 simulations. In the initial learning phase, the model adapted at a similar rate as the participants, making an average of 2.20 ($SD = 1.48$) unrewarded responses in the never-rewarded experiment and 2.02 ($SD = 1.23$) unrewarded responses in the rarely-rewarded experiment until its selection converged to the rewarded position. During reversal learning phases, it made significantly more unrewarded responses than the human participants in both experiments (never-rewarded: $M = 4.12$, $SD = 0.25$, two-tailed Welch's t-test, $t(68) = 14.04$, $p < .001$, $d = 6.89$, 95% CI 1.70 to 2.07; rarely-rewarded: $M = 4.51$, $SD = 0.2$, two-tailed t-test, $t(68) = 27.57$, $p < .001$, $d = 9.53$, 95% CI 1.75 to 2.01).

S 1.3 Cortico-basal ganglia plasticity

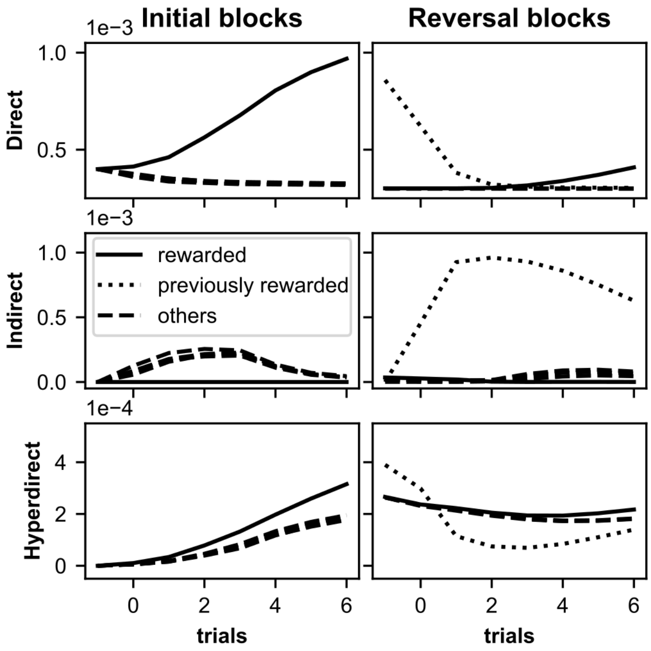

Figure S 3: The average synaptic weights of the direct, indirect and hyperdirect pathways (Cortex→StrD1, Cortex→StrD2, Cortex→STN), grouped according to the five sub-channels, related to Figure 3. Plotted data are the average of 60 initial blocks and 3540 reversal blocks from 60 simulations of the never-rewarded experiment. Following a rewarded response, LTP in the sub-channel of the selected response in the direct pathway promotes the reselection of this response. Similarly, hyperdirect pathway weights increase during rewarded responses, but for all sub-channels, resulting in local selection by the direct pathway and global surround suppression by the hyperdirect pathway. In the initial blocks, the indirect pathway is only weakly active as unrewarded responses cause dopamine undershoots. As the rewarded response changes, unrewarded responses lead to dopamine undershoots, causing the weights of the direct pathway to decrease. At the same time, the weights of the indirect pathway increase, mainly in the sub-channel of the previously rewarded response, suppressing its selection. When the newly rewarded response is discovered, the weights of the direct pathway in the corresponding sub-channel slowly increase again and those of the indirect pathway decrease. StrD1 – D1 dopamine receptor expressing striatal projection neurons, StrD2 – D2 dopamine receptor expressing striatal projection neurons, STN – subthalamic nucleus, LTP – long-term potentiation

S 1.4 Clockwise and counter-clockwise search

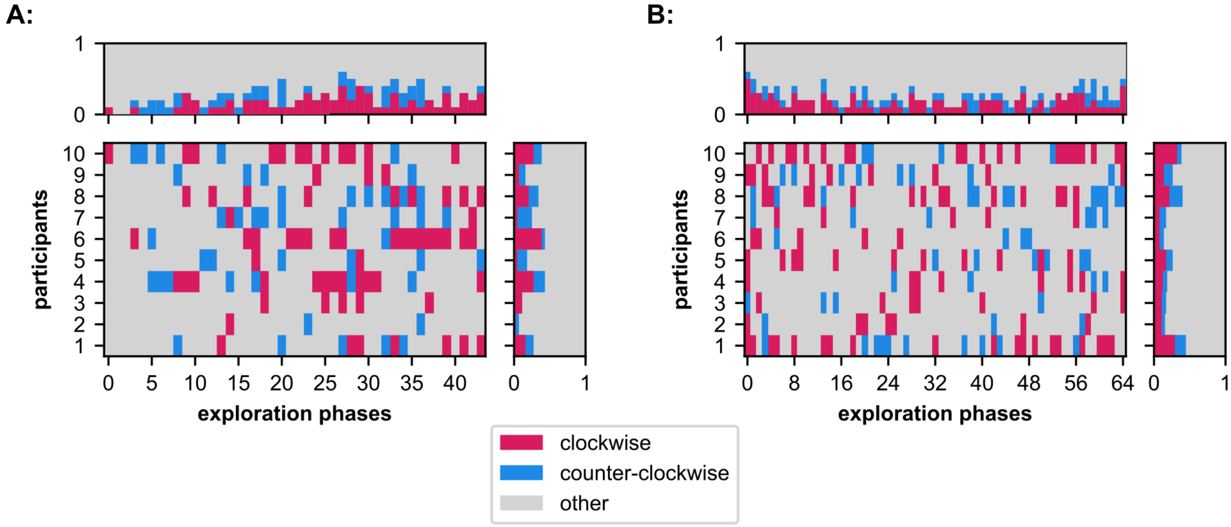

Figure S 4: The occurrence of clockwise and counter-clockwise search strategies, related to Figure 4. **(A):** Data from the never-rewarded experiment. The top histogram shows the frequencies of clockwise and counter-clockwise strategies for each exploration phase averaged over participants from the never-rewarded experiment. The right histogram shows the frequencies for each participant averaged over all exploration phases. The matrix plot shows which search strategy was used for each exploration phase and participant. **(B):** Data from the rarely-rewarded experiment presented as in A.

S 1.5 Progress of the number of never-rewarded responses

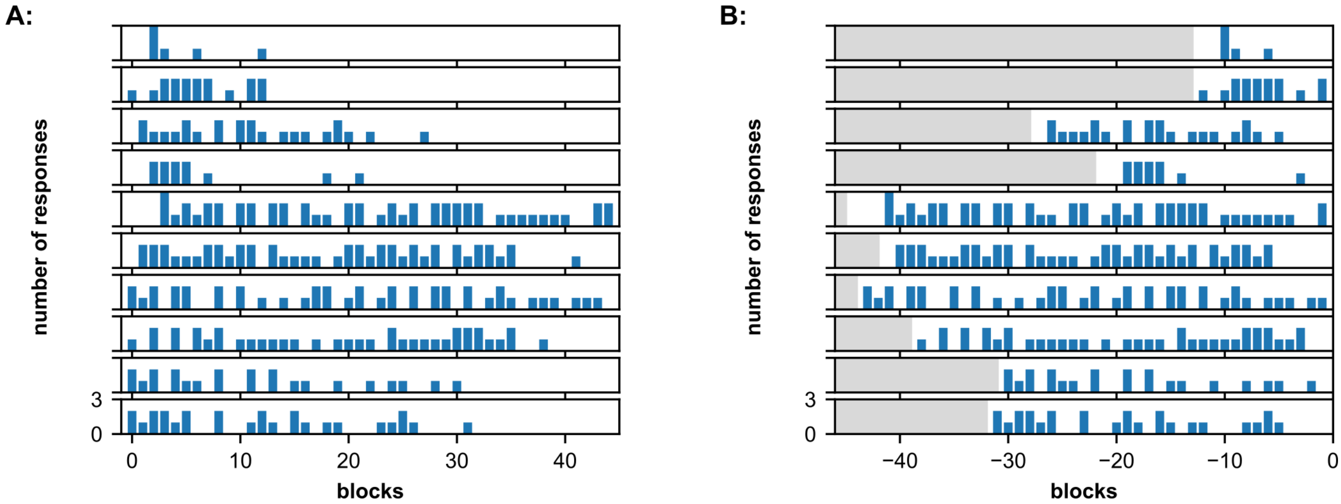

Figure S 5: Number of never-rewarded responses, related to Figure 5. **(A):** The distribution of never-rewarded responses for each participant (one row for each participant) shows that participants stopped choosing the never-rewarded positions at different points in the experiment. **(B):** Same data centered on the block in which participants chose a never-rewarded position for the last time. Blocks where no data are available after realignment are highlighted in grey.

S 1.6 Additional model exploration behavior results

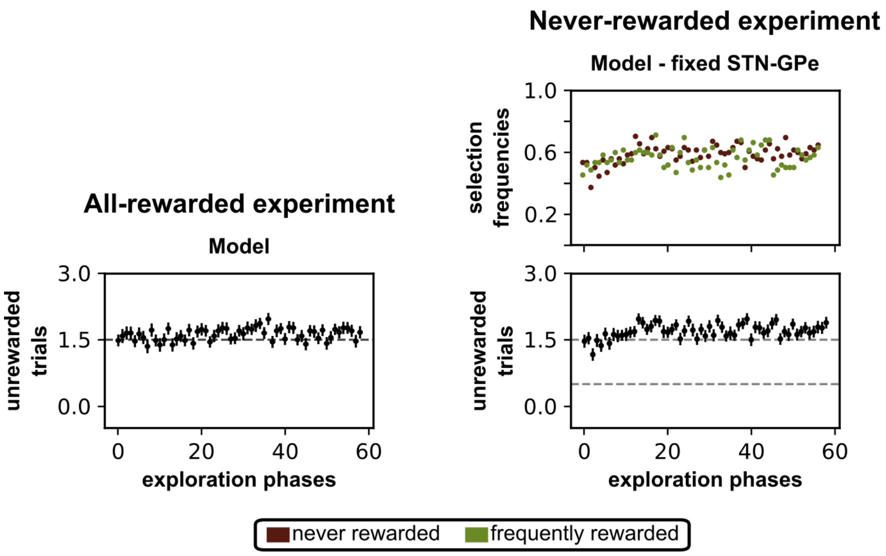

Figure S 6: Selection frequencies of unrewarded exploration trials of the model for the all-rewarded experiment with the fully learning model and the never-rewarded experiment with the fixed STN-GPe model, related to Figure 4. The data are presented as in Figure 4 of the main text. The top row shows the selection frequencies of never and frequently rewarded responses, and the bottom row shows the mean number of unrewarded exploration trials with SEM as error bars. Data for each experiment were obtained from 60 simulations. In the all-rewarded experiment, all positions are equally frequently rewarded (as in the participants' familiarization phase). The selections cannot be divided into rarely and frequently rewarded, but the number of unrewarded exploration trials does not change, showing that there is no change in exploration behavior. Accordingly the learning rate is not significantly larger than zero (M = -0.07, SD = 0.31, one-tailed t-test for one sample: $t(59)$ = -1.76, p = .958, d = -0.23, 95% CI -0.15 to 0.01). In the never-rewarded experiment with the model using the fixed STN→GPe projection, no bias develops (start: $\tilde{\chi}^2(1) = 0.44$, p = .508; mid: $\tilde{\chi}^2(1) = 0.26$, p = .611; end: $\tilde{\chi}^2(1) = 0.01$, p = .908, $\alpha_{corrected} = .01\overline{6}$, Bonferroni corrected for three tests) and thus the number of unrewarded exploration trials does not decrease (i.e. the learning rate is not significantly larger than zero, M = -0.16, SD = 0.29, one-tailed t-test for one sample: $t(59)$ = -4.14, $p > .999$, d = -0.54, 95% CI -0.23 to -0.08). SEM – standard error of the mean, STN – subthalamic nucleus, GPe – external globus pallidus

S 1.7 Original model weight changes

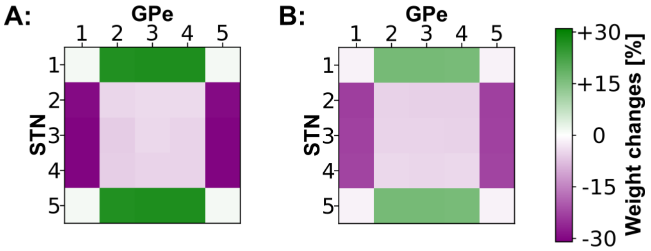

Figure S 7: Learned connectivity pattern in the STN→GPe projection of the original model for the never-rewarded **(A)** and rarely-rewarded **(B)** experiment, related to Figure 6. The data are presented as in Figure 6 (C and D) of the main paper. For each experiment, data are obtained from 60 simulations. In both experiments the cluster connectivity pattern develops. STN – subthalamic nucleus, GPe – external globus pallidus

S 1.8 Ideal observer decision tree

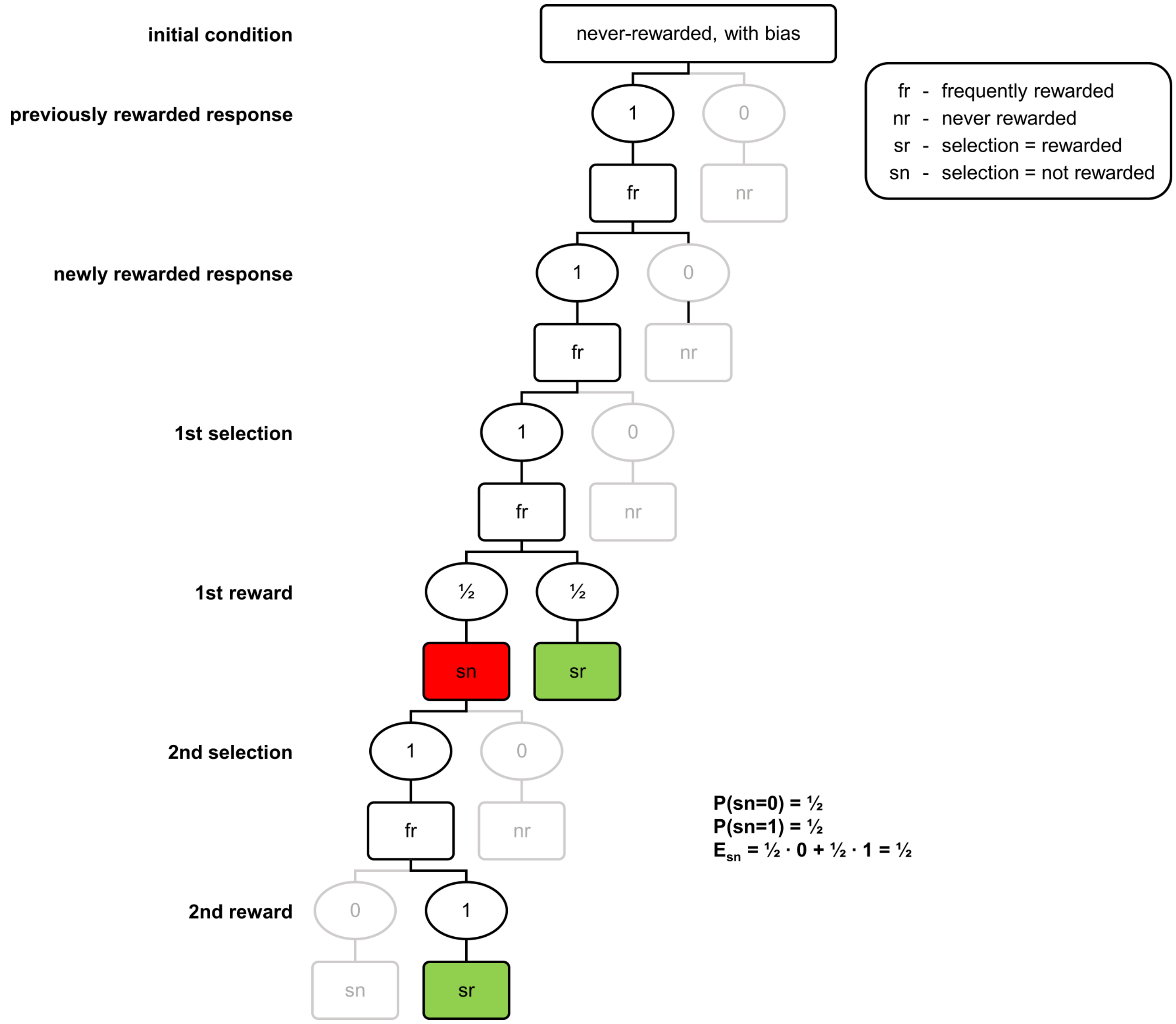

Figure S 8: Decision tree example of the ideal observer, related to STAR Methods. The top box describes the version of the experiment and whether the observer is subject to the exploration bias. This determines the probabilities of the previously and newly rewarded positions being never or frequently rewarded, the reward probabilities of the selections of the never and frequently rewarded positions, and the observer's selection probabilities. The tree from top to bottom describes: whether the previously rewarded position is never or frequently rewarded, whether the newly rewarded position is never or frequently rewarded, whether the observer's first selection is a never or frequently rewarded position, whether the selection is rewarded or not, and so on until no more selections are possible. The probabilities are shown in the ovals, the resulting events in the boxes. The decision tree shows that there are two possible decision paths. These two decision paths give the expected value for the number of selections not rewarded (i.e. the expected number of unrewarded exploration trials).

S 1.9 Learning rates from exponential fits

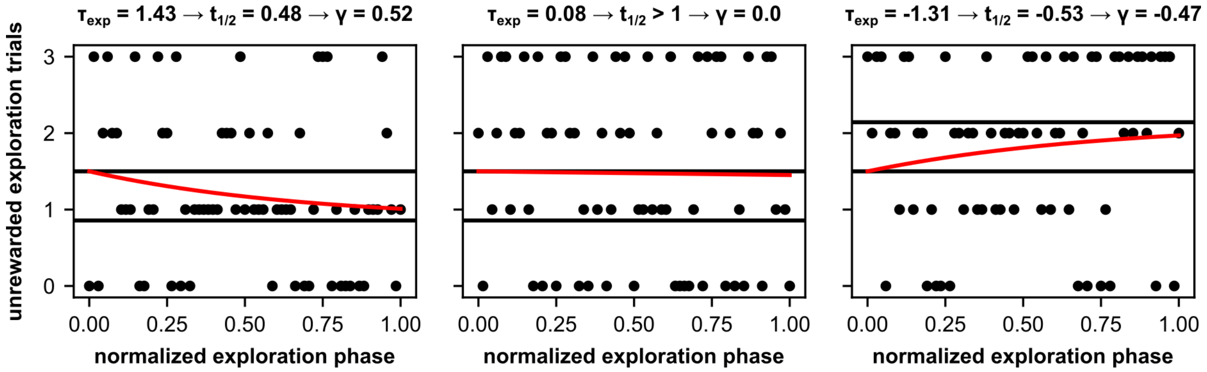

Figure S 9: Three examples of learning rates obtained from exponential fits, related to STAR Methods. The data shown are taken from 3 model simulations of the rarely-rewarded experiment. The raster plots show the number of unrewarded exploration trials for each exploration phase, where the exploration phases are normalized (divided by the maximum exploration phase). The two horizontal black lines indicate the start (x = 0) and end (x→ ∞) values of the fitted exponential curve. The start and end values are determined by the expected values for an ideal observer (with and without exploration bias). As can be seen in the example on the right, the exponential function is also allowed to increase symmetrically to the start value (then the end value is above the start value by the same absolute distance). The time constants of the fitted exponential function (τ_{exp}), the resulting half-life ($t_{1/2}$) and the resulting learning rate (γ) are given above the respective figures.

S 2 Supplemental tables

Population	Str	STN	GPe	SNr	SC	SC-I
C [pF]	50	1	1	1	1	1
$n2$ [nS mV^{-1}]	1.14	0.04	0.04	0.04	0.04	0.04
$n1$ [nS]	129.73	5	5	5	5	5
$n0$ [pA]	3082.56	140	140	140	140	140
v_r [mV]	-80	0	0	0	0	0
a [ms^{-1}]	0.05	0.005	0.005	0.005	0.02	0.1
b [nS]	-20	0.265	0.585	0.585	0.25	0.2
c [mV]	-55	-65	-65	-65	-65	-65
d [pA]	377	2	4	4	0.05	2
v_{peak} [mV]	40	30	30	30	30	30
I_{base} [pA]	0	0	0	10	0	0

Table S 1: Parameters for the neuron models of the different populations, related to STAR Methods. The units of the variables are given in brackets. As mentioned in the main text, the parameters are taken from different previous modeling studies [1–3]. The slightly different forms of the neuron models were converted here into a uniform form, whereby also some parameters were converted. Thus, the exact parameter names and values of the striatal cells differ from the previous ones for some parameters.

Target population	Weight [nS]	Poisson firing rate [Hz]
StrD1 (exc)	0.75	25
StrD1 (inh)	0.75	25
StrD2 (exc)	0.75	25
StrD2 (inh)	0.75	25
STN	0.19	100
GPe	0.015	100
SNr	0.11	100
SC	0.005	100

Table S 2: Parameters for the Poisson-train external inputs of the neurons of the different populations, related to STAR Methods. The units of the variables are given in brackets. For striatal neurons the presynaptic Poisson neurons provide both excitatory (AMPA synapse) and inhibitory (GABA synapse) input. For all other populations the Poisson neurons only provide excitatory input.

Parameter	Cortex-StrD1	Cortex-StrD2	Cortex-STN	Cortex-SC
α	0.15×10^{-3}	-0.045×10^{-3}	0.7×10^{-3}	-
Δ_{PRE}	0.8	0.2	0.03	1.8E^{-}6
Δ_{POST}	0.1	0.006	0.02	2E^{-}7
τ_E [ms]	120	1600	90	-
τ_{PRE} [ms]	300	450	30	30
τ_{POST} [ms]	20	20	20	3
τ_{DA} [ms]	150	300	50	-
δ [nS]	5E^{-}5	25E^{-}5	0	6E^{-}7
Min w [nS]	0.015	0	0	0
Max w [nS]	0.05	0.055	0.0005	6E^{-}6
Max E	2.5	8	-	-
Max A_{PRE}	1.5	-	-	-
w_{INIT} [nS]	$\mathcal{N}(0.02, 0.002)$	0	0	0

Table S 3: Parameters for the plastic cortical projections, related to STAR Methods. The units of the variables are given in brackets. The initial weights of the Cortex-StrD1 projection are drawn from a normal distribution $\mathcal{N}(\mu, \sigma)$ with mean μ and standard deviation σ. StrD1 – D1 dopamine receptor expressing striatal projection neurons

Presynaptic	Postsynaptic	Prob. same	Prob. diff	Weight [nS]
StrD1	SNr	0.35	0.0	0.0085
StrD2	GPe	0.25	0.0	0.005
STN	SNr	0.3	0.0	0.0008
GPe	SNr	0.35	0.0	0.0055
SNr	SC	0.35	0.0	0.0007
SC	StrD1	0.4	0.0	0.05
SC	StrD2	0.4	0.0	0.05
SC	SC-I	0.35	0.0	0.0025
SC-I	SC	0.0	0.35	0.0008
StrD1	StrD1	0.0	0.35	0.75
GPe	STN	0.35	0.05	0.007
STN	GPe	0.05	0.3	0.00063*

Table S 4: Probabilities and weight values used in each fixed projection, related to STAR Methods. For each projection, the table shows both the probability of creating a synapse between neurons which belong to the same sub channel and a different sub channel. The * for the STN→GPe projection indicates, that its synapses are plastic and here the initial weights are given. STN – subthalamic nucleus, GPe – external globus pallidus

References

[1] Humphries, M. D., Wood, R., and Gurney, K. (2009) Dopamine-modulated dynamic cell assemblies generated by the gabaergic striatal microcircuit. *Neural networks*, **22**, 1174–1188.

[2] Thibeault, C. M. and Srinivasa, N. (2013) Using a hybrid neuron in physiologically inspired models of the basal ganglia. *Frontiers in computational neuroscience*, **7**, 88.

[3] Izhikevich, E. M. (2003) Simple model of spiking neurons. *IEEE Transactions on neural networks*, **14**, 1569–1572.
